# An ethnopharmacological approach to evaluate antiparasitic and health-promoting abilities of *Pueraria tuberosa* (Willd.) DC. in livestock

**DOI:** 10.1371/journal.pone.0305667

**Published:** 2024-07-19

**Authors:** Suman Kalyan Mandal, Niladri Mukherjee, Anindya Sundar Ray, Samik Hazra, Sathi Saha, Swetarka Das, Nikhilesh Joardar, Saradindu Saha, Santi Prasad Sinha Babu, Chowdhury Habibur Rahaman

**Affiliations:** 1 Ethnopharmacology Laboratory, Department of Botany, Visva-Bharati, Santiniketan, India; 2 Parasitology Laboratory, Department of Zoology, Visva-Bharati, Santiniketan, India; 3 Division of Microbiology, CSIR-Central Drug Research Institute, Lucknow, Uttar Pradesh, India; 4 Department of Biotechnology, Indian Institute of Technology Kharagpur, Kharagpur, India; University of Milan, ITALY

## Abstract

In eastern India, the tubers of *Pueraria tuberosa* (Willd.) DC. are used by the ethnic communities for its wide range of medicinal and nutritional value, especially to rejuvenate livestock health and to treat helminthiasis. The study is aimed to evaluate the ethnoveterinary medicinal importance of *P*. *tuberosa* as anthelmintic, to verify its nontoxic nature and identify the most potent phytoconstituents aided by *in silico* molecular docking technique. Ethnomedicinal data collected from 185 informants were quantitatively analyzed employing eight quantitative indices to highlight the use diversity and most frequently used part of the plant. High scores of certain indices employed, such as Use Value (UV = 0.52), Fidelity Level (FL = 68.42%) and Tissue Importance Value (TIV = 1) clearly illustrate an ethnomedicinal lead regarding medico-nutritional benefits of the tuber part used against intestinal helminthic diseases of veterinary animals. Based on this ethno-guided lead, root tuber has been investigated for its chemical profiling by the estimation of total phenolics, flavonoids, tannins and alkaloids, along with HPLC and GC-MS analyses. Anthelmintic property was evaluated with the tuber extracts by *in vitro* studies on some helminths of livestock and poultry birds, and it showed promising results against the tested parasites namely *Cotylophoron cotylophorum*, *Raillietina tetragona* and *Setaria cervi*. Toxicity assessments of tuber extract through *in vitro* and *in vivo* methods were performed using Vero cells and BALB/c mice. Nontoxic nature of the studied tuber extract was observed even in higher experimental doses. Out of 12 phytocompounds identified by GC-MS analysis, one compound [Morphinan-4,5-epoxy-3,6-di-ol,6- (7-nitrobenzofurazan-4-yl) amino-] exhibited the best binding conformations in cost of the lowest binding energy values with six target proteins that include one anti-inflammatory, one antioxidant, and four anthelmintic proteins. The findings of our study are found very encouraging to evaluate this tuber drug furthermore intensively towards the development of anthelmintic veterinary medicine.

## 1. Introduction

From the onset of human civilization, livestock has been mainly reared for companionship, labour, food, and secondary products. The prevalence of several parasitic diseases in veterinary animals puts their valuable and profitable services at high risk. Amidst various disease restrictions that impact the livelihood of marginal farmers and livestock rearers, gastrointestinal parasites of livestock emerged as the most prevalent global index [1–3]. It is quite concerning that farm animals are becoming resistant to anthelmintic medications due to overuse and inadequate formulations of most synthetic pharmaceuticals [4]. Hence, there is an urgent and ubiquitous need to control infections caused by the intestinal parasites in all types of ruminants. An integrated approach is highly demanded for sustainable control of intestinal parasites and to overcome anthelmintic resistance in livestock. This integrated approach of parasitic disease management embodies biological control, reduced frequency of synthetic anthelmintic medicaments, parasite vaccines, livestock breeds resistant to parasites, use of plants with anti-parasitic properties, and the use of traditional herbal remedies. Ethnopharmacological leads can play a central role in this integrated approach of finding novel anthelmintic drug substances [5, 6]. Significant numbers of ethnomedicinal plants have so far been reported as anthelmintic agents from different parts of the world, including India [7–10].

Indian Kudzu or *Pueraria tuberosa* (Willd.) DC. is a leguminous woody climber with significant economic importance, and it is used as one of the major constituents in several Ayurvedic formulations. In different parts of India, the underground tuber of this plant is used by the traditional people to alleviate sexual weakness, diarrhoea, fever, chest pain, rheumatism, inflammations, diabetes, and skin problems in humans [11, 12]. The tuber of *P*. *tuberosa* is also used effectively as ethnoveterinary medicine in different states of India to treat several health disorders like urinary trouble, retention in milk secretion, tuberculosis, dyspepsia, and helminthiasis in livestock [8, 13–15].

It is commonly noticed that there is an extensive use of medicinal plants, but very little work has been done to establish their safety and efficacy. It has been reported from different scientific research that many medicinal plants like *Callilepis laureola*, *Flabellaria paniculata*, *Tephrosia vogelii*, *Uncaria tomentosa*, and many others used for curing a wide range of diseases are potentially toxic, carcinogenic, or mutagenic when they are administered at high doses for a prolonged period [16–18]. It is expected that all the drugs prescribed for therapeutic purposes should be efficacious, reliable, consistent, and safe from any toxicological hazards. Information regarding the toxicity profile of medicinal plants should be gathered to achieve assurance of their safe use for humans and domesticated animals and definitely for the development of effective pharmaceuticals [19]. The scientific community must thus carefully assess the toxicity risk of traditional herbal remedies in addition to their pharmacological and phytochemical screening in light of the safety concerns.

*P*. *tuberosa* has been studied extensively for its wide array of potentiality as an immune booster, restorative tonic, antiaging, galactagogue, spermatogenic, demulcent, purgative, nutritive, and antioxidant [11, 12, 20, 21]. Though the tuber of this Ayurvedic plant has immense medicinal importance, no scientific studies and *in silico* analyses have been done to examine its phytoconstituents for veterinary anthelmintic activity. In this context, a set of tests using experimental and computational analysis were carried out to scrutinize and evaluate the anthelmintic potentiality of *P*. *tuberosa* tuber.

The present study aims to document the ethnomedicinal uses of *P*. *tuberosa* from eastern India as well as evaluates the anthelmintic potentiality of the plant along with its phytochemical profiling, pharmacological investigation, toxicity assessment, and identifying the possible phytocompounds of anthelmintic potentiality in conjunction with its mechanism of action through *in silico* models.

## 2. Materials and methods

### 2.1. Study area

The Eastern region of India mainly comprises the states of Bihar, Jharkhand, Odisha and West Bengal. The laterite region of West Bengal is mainly situated towards its western part, and it is the protracted part of the eastern fringes of the Chota Nagpur plateau covering an area of approximately 7,700 km^2^ which represents 22.3% of the total geographical area of the state of West Bengal. The percentage of Scheduled Tribes in this zone is 11.85% and the Scheduled caste is 26% [22, 23]. Some of the tribal groups in this region are Santal, Kora, Munda, Bhumij, Kharwar, Mal-Pahariya, Oraon, Savar, Birhore, Kherwal, Ho, Mahali and others.

**Fig 1:**
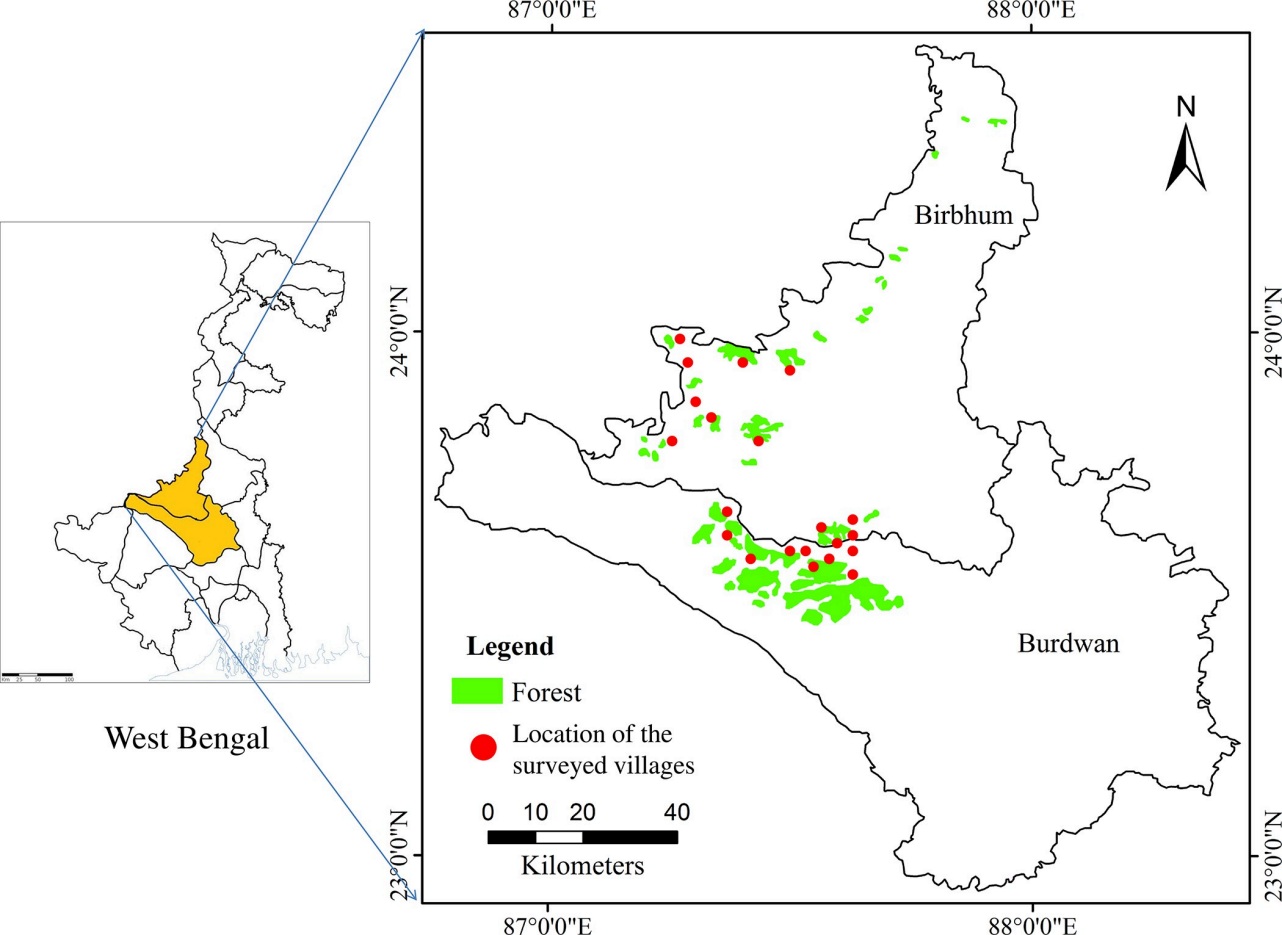
Study area.

The study area of present work is delimited in the northern part of the laterite zone of West Bengal, which includes mainly the recently formed Paschim Bardhaman district (Paschim Bardhaman and Purba Bardhaman were formed on 7 April 2017 after bifurcation of the previous Burdwan district of West Bengal) and northern, western, and south-central parts of Birbhum district (Fig 1). This part of the laterite region covers approximately 2290 km^2^ which represents 29.74% of the total laterite cover of West Bengal. Altogether 21 villages have been selected for the present study which include 12 villages from Birbhum district and nine from Pashchim Burdwan district.

### 2.2. Interview and questionnaire

The survey was conducted for two years (May 2016 –April 2018) to collect information regarding the ethnomedicinal uses of *P*. *tuberosa*. Before data collection, prior informed consent (PIC) was taken from the informants. For the collection of data, the best field practice as critically described earlier was followed, and the Code of Ethics mentioned by the International Society of Ethnobiology was also consulted [24, 25].

The data regarding ethnomedicinal uses of *P*. *tuberosa* were collected from elderly well-informed persons by interviewing them with the help of a semi-structured open-ended questionnaire [26]. Most of the informants are under the low-income group, and they are attached to different socio-professional designations like traditional healer of livestock diseases (Go-Vaidya), herbalist, exorcist, and shephards. Information regarding knowledge of the plant, local name, ethnobotanical uses, season and mode of collection, preservation, remedy preparation and administration, dosage, the market value of the plant, and socio-demographic features of the informants were collected.

### 2.3. Quantitative indices for ethnobotanical data analysis

Data recorded from the field survey on traditional uses of *P*. *tuberosa* was analyzed with the following quantitative indices:

- Use Knowledge Index (UKI) was employed to analyze the level of novelty in local names not yet documented and to appraise the continued use of plants in ethnomedicine [27]. UKI = ΣU/K, where ‘U’ represents a respondent who uses *P*. *tuberosa* and ‘K’ is the respondent who knows the plant by names like “Bhuin-Kumro” or “Patal-kondha” or others.
- Ethnobotanical Richness (ER) was adopted as a property tool to quantify the ethnic group-specific application of *P*. *tuberosa*. ER = E_r_/n, where ‘E_r_’ is the respondents who have recognized the species as a medicinal plant and ‘n’ represents the total number of respondents [28].
- Use Value Index (UV) was employed to determine the level of importance attached to the *P*. *tuberosa* plant used by the respondents [29]. UV = ΣU/n_s_ where ‘U’ is the number of uses mentioned by a respondent, ‘n_s_’ is the number of respondents who use medicinal plant preparations for any purpose.
- Fidelity Level (FL) suggested by Friedman et al. was applied here to quantify the percentage of respondents claiming the use of *P*. *tuberosa* for the same major purpose [30]. FL(%) = N_p_/n × 100, where ‘N_p_’ is the number of respondents claiming a specific use for *P*. *tuberosa* and ‘n’ is the total number of respondents using the plant species for any purpose.
- Knowledge Value Index (KVI) helps to evaluate the level of knowledge of particular plant species and further appraises the extent of awareness of ethnomedicinal plants among a population without paying special attention to the names of plant species [31]. KVI = ΣA/n, where ‘A’ represents a respondent awares or knows the plant *P*. *tuberosa* without necessarily knowing the plant by common name/botanical name; ‘n’ is the total number of respondents interviewed.
- Ethnomedicinal Income Index (EI) was used to quantify the relative ethnomedicinal economy of a local population based on local knowledge of plant use [31]. EI = ΣI/n, where ‘I’ represents respondents who use this plant species for any given purpose.
- Tissue Importance Value (TIV) is applied to identify the most useful part of the plant *P*. *tuberosa* to the local people [31]. TIV = N_t_/n, where ‘N_t_’ is the interviewee who mentioned a particular part of *P*. *tuberosa* for use and ‘n’ is the total number of respondents who use the plant for any purpose.
- Name Homogeneity Index (NHI) was used to quantitatively describe the variation in names used by respondents to describe *P*. *tuberosa*. Differences exist in local terms used to describe or identify the medicinal plants, especially in a multi-ethnic settlement [31]. NH = Nh/n, where ‘Nh’ is the number of similar names known to the interviewee, and ‘n’ is the total number of the interviewee who knows the plant in any of their local/tribal names.

### 2.4. Chemicals and reagents

Milli-Q water (Milli-Q Academic with 0.22μm Millipak R-40) was used for all the assays. Antibiotics, foetal bovine serum (FBS), gallic acid, pilocarpine, 2,2-diphenyl-1-picrylhydrazyl (DPPH), tetracycline, streptomycin, griseofulvin, gallic acid, naringin, catechin, quercetin, and curcumin were obtained from Sigma-Aldrich Co. (St. Louis, MO, USA). MTT (3-(4, 5-dimethyl-thiazol-2-yl)-2, 5-diphenyl-tetrazolium bromide), RPMI-1640, ethanol, methanol, ethyl acetate, chloroform, hexane, acetone, Folin-Ciocalteu’s phenol reagent, NaOH, KOH, HCl, 1,10 phenanthroline, sodium carbonate anhydrous, and ammonium molybdate, were purchased from Hi-Media Laboratories, Mumbai (India).

### 2.5. Collection of plant part, identification and preservation of the plant specimen

**Fig 2:**
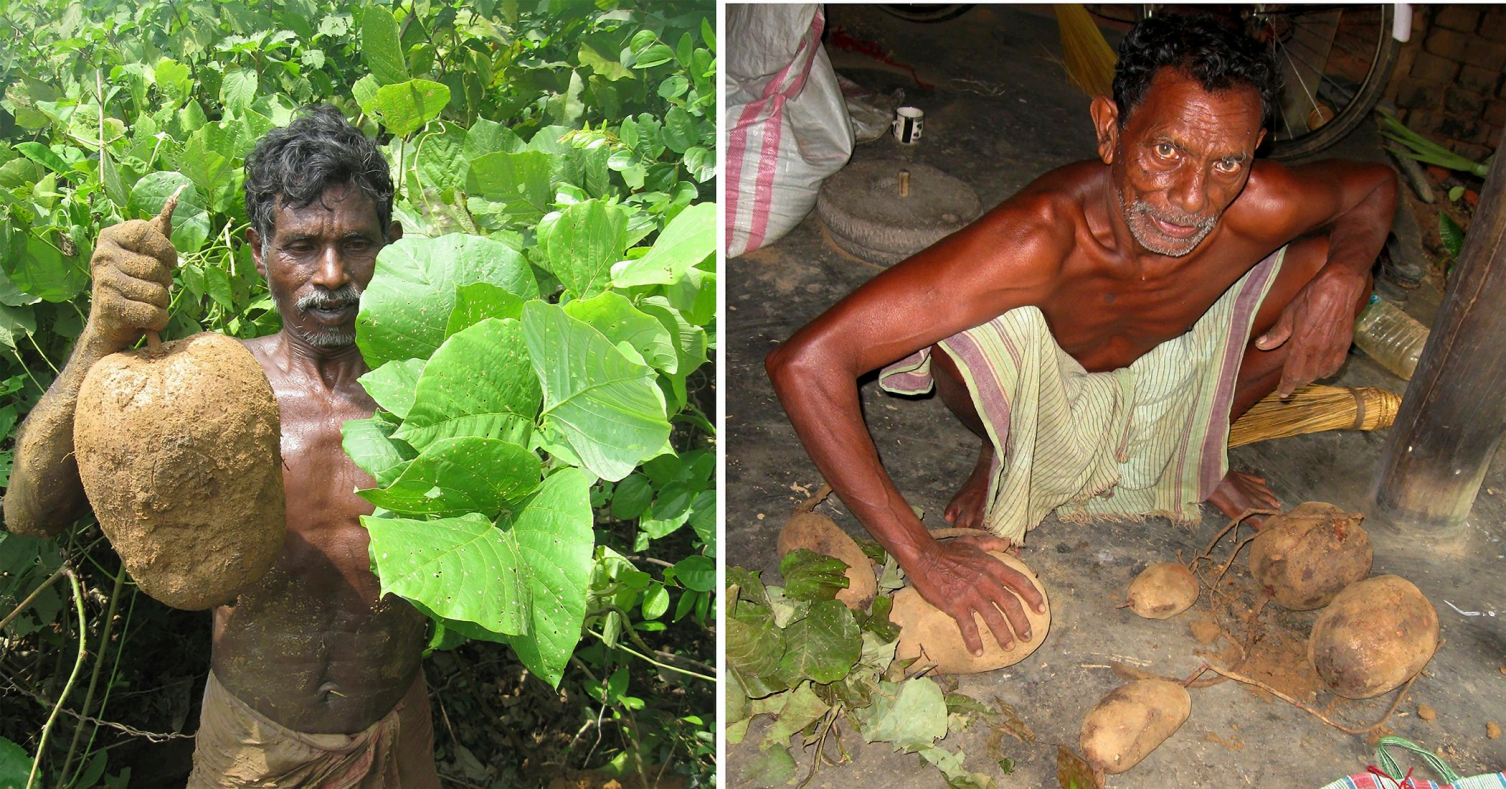
Collection of the tuber of *P*. *tuberosa*; two tribal medicine men (Mohon Soren from Arjuri, Burdwan and some Baskey from Khayerdanga, Birbhum) are helping in collecting and gathering the tubers.

Mature underground root tubers of *P*. *tuberosa* (from 1.5 kg to 5 kg of weight) were harvested in September 2016 (when the flowering and fruiting season is over; collection date- 21.09.2016) from an open forest fringe by a river-bank near Aarjuri village of Paschim Burdwan district, West Bengal (23°31ʹ56.4ʺ N and 87°30ʹ36.8ʺ E) following the guideline set by the National Medicinal Plants Board, India [32]. The collected plant species (Fig 2) were identified with the help of Flora of West Bengal [33] and a plant taxonomist. The nomenclature of the identified plant species has been updated following the standard website Plants of the World Online [34]. The collected plant specimen has been preserved following standard herbarium techniques [35] and kept in the Department of Botany, Visva-Bharati, Santiniketan, India for future references [Voucher specimen number: INDIA, West Bengal, Burdwan district, Aarjuri, 21.09.2016, SK Mandal 274 (VBH)].

### 2.6. Preparation of plant extracts

The collected plant part was washed thoroughly, sliced into several thin pieces, shade dried, and grounded into a fine powder. The plant powder was then preserved in an airtight container at 4°C for future use.

*P*. *tuberosa* tuber powder (10 gm) was extracted with 100 mL of different solvents (water, ethanol, methanol) separately in a ratio of 1:10 (plant powder: solvent) in a 250 mL conical flask and kept in a mechanical shaker in continuous agitation for 36 h (28±2°C temperature is maintained). The whole process was repeated thrice for single extraction. The slurry substance thus obtained was filtered with Whatman’s (No.1) filter paper. The filtrate was concentrated by evaporation at room temperature (28±2°C). The ultimate yield was stored at -20°C and dissolved in Dimethyl sulfoxide (DMSO) to make the stock before use.

### 2.7. Quantitative phytochemical screening

Total phenolic content was estimated following the standard method [36]; total flavonoid was estimated employing the aluminium chloride method [37]; the method of Afify et al. [38] with slight modifications was followed for total tannin content estimation and total alkaloid content was measured using 1,10-phenanthroline method described by Singh et al. [39] with slight modifications.

For HPLC analysis, 500 mg of each plant sample was macerated with 20 mL HPLC grade solvents (water, ethanol, and methanol; purchased from Merk, India) were used as solvents for maceration in separate experimental set up for 36 h with continuous shaking. The extracts were filtered through a 0.22 μm syringe filter and 20 μL of the extract was injected for the HPLC analysis. The extracts were analyzed in HPLC (ThermoFisher Scientific Dionex Ultimate 3000, Germany; with a quaternary solvent delivery system (LPG 3400 SD) having a diode array detector, DAD 3000). Each compound was detected by its retention time and by spiking with standards under the same conditions. The quantification of the sample was made by the measurement of the integrated peak area. Chromeleon 6.8 software was used to process the data [40]. The data were reported with the convergence limit in triplicate.

For GC-MS analysis, at first 5 gm of dried plant powder was saturated in 100mL of methanol for extraction. The solution was subjected to occasional stirring for 72 h and the extract was then filtered using Whatman No.41 filter paper. Then the crude extract was completely concentrated by evaporating the methanol in a rotary evaporator at 40°C. Thereafter, 0.1 gm of the dried, concentrated extract was dissolved in 10 mL methanol. The stock solution of extract was then transferred to an air-tight container and kept in the refrigerator at 4°C until required for the experiment.

Gas Chromatography-Mass Spectrometry (GC-MS) analysis was carried out on a Mass spectrometer (GCMS- model number Agilent 6890-MS). The sample solution was taken for GC-MS analysis after filtering it in a sterilized millipore filter (0.22 μ). 1μL of the sample was injected into a GC equipped with an MS and a non-polar capillary column HP-5 (30 m × 0.25 mm; 0.25 μm).

The phytochemical constituents present in the plant sample was identified by consulting the mass spectral library of NIST (NIST ver. 2.0, 2005). The NIST database was thoroughly checked, extracted the spectra of reference compounds, and it was matched with the unknown phytochemical compounds present in the drug sample. Percentages of peak areas of the individual chemicals were determined with respect to the total peak area of all chemical compounds detected from the chromatogram.

### 2.8. Proximate and mineral content analysis

Proximate content (moisture, ash, crude protein, crude fat, crude fiber, and nitrogen-free extracts) and dietary mineral (Mg, Ca, Zn, Mn, Cu, Fe, Na, and K) content of the powdered samples was carried out following the standard methods [41–44].

### 2.9. Pharmacological investigations

#### 2.9.1. Antioxidant activity

ABTS and DPPH radical scavenging activity was determined following the standard method of Thaipong et al. [45].

#### 2.9.2. Anthelmintic activity

For the anthelmintic activity study, rumen was collected from freshly slaughtered goat from a local slaughterhouse and was washed properly in tap water. Two distinct-sized trematode parasites (*Cotylophoron cotylophorum*) were identified in different locations of the rumen. The larger type resides in the deeper region, whereas, the smaller ones present in the upper surface of the rumen. Parasites were carefully scratched out and separated according to their size and were kept in physiological saline in CO_2_ incubator (5.0% CO_2_) at 37°C.

From a local slaughterhouse, intestine of indigenous breed chicken was collected and pale white colored cestode, *Raillietina tetragona* was collected carefully, washed thoroughly with normal saline and incubated with normal saline in CO_2_ incubator at 37°C.

Filarial nematode parasites of cattle (*Setaria cervi*) were collected from an nearby abattoir and brought to the laboratory in a sterile container deeped in normal saline, were washed repeatedly with the same solution and kept in RPMI-1640 media at 37°C. Microfilaria (Mf) were collected from gravid females and kept in RPMI-1640 media at 37°C.

Collected helminth parasites like *Cotylophoron cotylophorum* (Rumen fluke), chicken cestode, *Raillietina tetragona* (Oval-suckered tapeworm), and adult and mf of filarial nematodes, *Setaria cervi* (Bovine filarial roundworm) were incubated with different concentrations of aqueous, ethanolic and methanolic extracts of *P*. *tuberosa* tuber for 24 h at 37°C, 5.0% CO_2_ in sterile fiber petri-plates (60 mm diameter, Tarson, India) with 10 mL complete RPMI-1640 (10% FBS) for all the parasites except mf of *S*. *cervi* which were maintained in 24 well plates with final media volume of 2 mL. The experiments were executed in triplicate and repeated at least four times.

For the determination of parasite mortality by MTT assay and LC_50_ determination of exposed parasites, viability of all the exposed parasites was determined by the MTT [3-(4,5-dimethyl–thiazol-2-yl)-2,5-diphenyl-tetra-zoliumbromide] reduction assay at the cellular level following conventional technique and modifications applicable for helminth parasites [46, 47]. All other parasites were collected and incubated with MTT after taking the weight. The percentage mortality evaluation was performed by calculating the reduction in the formation of formazan crystals concerning the unit weight of the exposed parasites. Additionally, mf of *S*. *cervi* viability was also assessed by Trypan blue that selectively stains dead parasites.

During the microscopy of exposed parasites, micrographs of control and treated parasites were taken under an inverted microscope (Dewinter, Italy). Both bright field and phase-contrast micrographs of the nematode mf were taken to notice morphological aberrations if there are any. For the trematodes and cestodes, possible morphological alterations were noticed after exposing them with Trypan blue and with the expectation that it will selectively stain the dead parts of the exposed parasites to observe possible morphological alterations.

### 2.10. *In silico* molecular docking study

The molecular docking of the phytocompouds identified in GC-MS analysis of the tuber extract of *P*. *tuberosa* was studied for their possible anti-inflammatory, antioxidant, and anthelminthic properties. The structure of the compounds were retrieved as sdf files from the Pubchem database (https://pubchem.ncbi.nlm.nih.gov/) and OPEN BABEL software (https://openbabel.org/wiki/Main%20Page) was used to convert them into Mol, PDBQT, and PDB file formats. The three-dimensional (3D) structures of the phytocompounds were optimized for molecular docking study using UCSF Chimera 1.15 software. The structure-based molecular docking was performed to study the protein-ligand interactions of the phytocompounds with anti-inflammatory (Tnf alpha, PDB id: 2AZ5), antioxidant (Glutathione reductase, PDB id: 1BWC) and anthelmintic [e.g., Asparaginyl tRNA synthase (PDB id: 1X55), GABA-A receptor (PDB id: 6D6T), Glutathione S-Transferase I (PDB id: 1AXD) and glutamate-gated chloride channel (PDB id: 3RHW)] target proteins obtained from the Research Collaboratory for Structural Bioinformatics (RCSB) Protein Data Bank using the iGEMDOCK and AutoDock 4.2 program [48, 49]. Interacting protein residues forming hydrogen bonds with phytocompounds were visualized using PyMOL [50].

### 2.11. Toxicity analysis

For *in vitro* cytotoxicity assay, cell toxicity assay was performed against the Vero cell line (ATCC- CCL-81) using the MTT assay following the standard protocol [51].

The assay of *in vivo* acute toxicity was performed according to the Organization for Economic Cooperation and Development (OECD) guideline 423 [52]. Acute toxicity study was performed on 6- to 8-weeks-old BALB/c mice procured from the National Laboratory Animal Facility of the CSIR-Central Drug Research Institute, Lucknow (IAEC/2017/262 (F-262)/renew-O/dated 31.10.2017), and experiments with small laboratory animals were performed as per the guidelines of Committee for the Purpose of Control and Supervision of Experiments of Animals (CPCSEA), Govt. of India (1819/GO/Ere/S/15/CPCSEA). The mice were housed in adequately ventilated hygienic cubicles inside the animal house and kept under standard environmental conditions (23–25˚C, 12 h/12 h light/dark cycle).

In this study, a total of 24 male and female BALB/c mice of weighing between 20–22 gm were randomly divided into four experimental groups of 6 mice each (3 males and 3 females per group). After fasting overnight, the extract was administered to each treatment group at single doses of 1000, 2500 and 5000 mg/kg, respectively, by oral gavage. The control groups were treated with the same volume of distilled water. The mice were allowed free access to feed and drinking water. After dosing, all animals were observed individually for mortality and changes in general behavior during the first 30 min, then at 2, 4, 6, 10 and 24 hours following treatment. Symptoms of toxicity such as hypo-activity, piloerection, breathing difficulty, tremors, and convulsion were evaluated after administration of the various extract doses. During the remaining experimental period, the animal observation was performed at least once per day for the post-dosing period of 14 days. Body weights were measured at the initiation of treatment, and on days 4, 7, 11 and 14 after administration. All animal facility staff and associated researchers had followed required guidelines and having expertise in conducting animal research. No animals were euthanized during this experiment.

### 2.12. Statistical analysis

All the experiments were repeated at least three times and data were expressed as the mean± SEM. Statistical significance and differences among groups were assessed with One-Way analysis of variance (ANOVA) followed by Tukey’s test. P values ≤ 0.05 (*) or ≤ 0.01 (**) were considered as indicative of significance.

## 3. Results and discussions

### 3.1. Ethnobotanical data and its analyses

Altogether 185 people (115 males, 70 females, aged between 21–85 years) were interviewed in two languages, i.e., *Bengali* and *Santali*. Photographs of the living plant specimen, along with the tuber labelled with the common name “*Bhuin-kumro*”, were shown to each interviewee to confirm its identification. Among the interviewees, 125 persons continued the questionnaires; 95 informants were familiar with the plant *P*. *tuberosa*, and nearly 89.5% of them knew the plant by any of its local names. These 95 interviewees were considered key informants for the present study. A total of 18 types of ethnomedicinal uses of the plant *P*. *tuberosa* have been recorded from the studied area. Among the recorded 18 uses, 11 types of uses were documented as ethnoveterinary medicine. Four types of uses against diseases like cattle diarrhoea, skin disease of a bullock, swelling dewlap of cattle and intestinal worm of livestock have been found new to the existing information on ethnoveterinary medicine of *P*. *tuberosa* reported from different parts of India so far. In the studied area, cattle owner and ‘*Go-vaidya*’ have specified knowledge of using *Pueraria* tuber in the treatment of helminthiasis of livestock. Tuber is given orally to the affected mature cow in two ways. Freshly collected tuber is chopped finely (250 gm) and given along with paddy straw once a day for 7–21 days or sliced tubers (500 gm) are soaked in 5L rice gruel overnight and then given orally the whole thing in the next morning once a day for 7–21 days. All the detailed other ethnomedicinal data recorded in the present study has been provided in Table 1.

**Table 1:** Recorded ethnomedicinal uses of *P*. *tuberosa* and quantification of it’s Fidelity Level (FL) and Use Value (UV).

Local /tribal name	Diseases / conditions treated	Parts used, remedy preparation and mode of administration	Number of citation	FL (%)	UV	Previous report
Bhuin-kumro (B, S), Patal- kumro (B), Kanda palash (B); Patal-kondha (S), Tirra (S)	Retention of milk	Tuber; tuber of *P*. *tuberosa*, root of *Curculigo orchioides* Gaertn. and *Bombax ceiba* L. are pounded in cow milk in a ratio of 2:1:1 and the paste is given orally with warm milk twice daily up to 7 days to a nursing mother.	45	47.36	0.36	[107]
	Retention of milk in cattle^#^	Tuber; dried powder (50 gm approx.) is given with old molasses daily twice for a week to the milch cow.	30	31.58	0.24	[107]
	Sterility of heifer^#^	Tuber and seed; freshly collected tubers (250 gm) are sliced and given along with cattle feed in the morning once a day and continue till the onset of normal ‘heat period’.	20	21.05	0.16	[108]
		If the tuber is unavailable, the aforementioned preparation is modified by replacing the 250 gm of tuber with 10 gm of seeds.				
	Vomiting	Tuber and leaf; fine dust of dried tuber (5 gm) is given orally with a little amount of fresh ginger and a pinch of rock salt if someone feels upsetting of stomach. Simultaneously handful of leaves are crushed and sniffed in such condition.	15	15.79	0.12	[109]
	Abdominal pain of bullock^#^	Tuber; tuber of *P*. *tuberosa* and bark of *Dillenia pentagyna* Roxb. (2:1) are made into a paste along with 11 grains of black pepper; mixed in 500 mL of water and administered orally once a day in empty stomach for a week.	15	15.79	0.12	[109, 110]
	Body ache	Tuber; equal amount of fresh tuber of *P*. *tuberosa* and bark of *Shorea robusta* Gaertn. are made into paste; heated slightly and applied the lukewarm mixture topically on the affected body part twice a day for three days.	10	10.53	0.08	[109]
	Fever with trouble in respiration of bullock^#^	Tuber and leaf; tuber of *P*. *tuberosa*, root of *Amaranthus spinosus* L. and bark of *Terminalia arjuna* (Roxb. ex DC.) Wight & Arn. (2:1:1) are made into a paste along with 9 grains of black pepper and given orally in an interval of six hours in a day. Simultaneously smoke is created with the fresh leaves of *P*. *tuberosa* into the cowshed.	15	15.79	0.12	[109]
	Cattle diarrhea^#^	Tuber; tuber of *P*. *tuberosa* and root of *Justicia gendarussa* Burm.f. (in 5:1 ratio) are made into paste; mixed well in water and given orally in the first morning once a day for consecutive 5 days.	25	26.32	0.20	New report
	Rheumatoid arthritis of bullock (“*Shimola-rog*”)^#^	Seed and tuber; tuber bark of *P*. *tuberosa* (10 gm) and 13 grains of seeds of this plant pounded together in water and made into a paste; heated slightly with a pinch of turmeric and salt; applied the whole mixture topically on the affected leg thrice a day till the cure.	10	10.53	0.08	[111]
	Skin disease of bullock^#^	Seed; dried seed dust is boiled in coconut oil and applied topically on the affected part.	15	15.79	0.12	New report
	Swelling of dewlap of cattle^#^	Tuber; freshly collected tuber of *P*. *tuberosa*, rhizome of *Curcuma longa* L. (2:1) are made into paste with 3 pieces of fresh leaves of *Calotropis gigantea* (L.) Dryand and mixed with ‘*Nishadal*’ (Ammonium chloride); applied topically throughout the dewlap of affected cattle once a day for 5 days.	10	10.53	0.08	New report
	Retention of urine	Tuber; crushed fresh tuber (nearly 100 gm) is taken orally with one tea-spoon full of molasses in empty stomach once a day till the cure.	20	21.05	0.16	[110]
	Stomach ache	Tuber; after a night of fasting, one tea-spoon full of fine dust of dried tuber is taken orally along with a glass of fennel soaked water in the next first morning.	15	15.79	0.12	[112]
		1 gm of fennel is soaked in a glass of water overnight and taken in the next morning.				
	Loose motion	Tuber; one tea-spoon full of dried tuber dust of *P*. *tuberosa* is taken orally along with finely pounded two seeds of *Abrus precatorius* L. and 11 grains of black pepper once a day for three days.	20	21.05	0.16	[110]
	Haemorrhagic septicaemia (HS) of cattle^#^	Leaf; fully mature leaves are collected (9 in numbers) and made it sacred by holy chanting. Both sides of the leaves are then smeared with turmeric paste and coconut oil; fed one by one with an interval of 30 minutes. The whole procedure is repeated once a day for 5 days.	10	10.53	0.08	[113]
	Suppurating wound	Tuber; fully mature tubers are collected, washed thoroughly, sliced into several thin pieces and made it shade dry. These dried flakes of tubers are then burnt into ash; ash is mixed with coconut oil and applied as poultice on the wound at least twice a day till the cure.	20	21.05	0.16	[114]
	Post partum debility of cattle^#^	Tuber; freshly collected tubers (500 gm) are sliced into thin pieces; soaked in 2L of rice gruel overnight and given the whole thing in the next morning for 7 consecutive days.	35	36.84	0.28	[10]
	Promote health	Tuber; one table-spoon full of dried tuber dust of *P*. *tuberosa* along with the same quantity of dried ‘Satavari’- *Asparagus racemosus* Willd. is mixed well in a glass of lukewarm milk. One adult can take orally once in the night for at least 15 days.	25	26.32	0.20	[115]
	Intestinal worm of cattle^#^	Tuber; tuber is given orally to the affected mature cow in two ways-	65	68.42	0.52	New report
		Freshly collected tuber is chopped finely (250 gm) and given along with paddy straw once a day for 7–21 days. Or				
		Sliced tubers (500 gm) are soaked in 5L rice gruel overnight and then given orally the whole thing in the next morning once a day for 7–21 days.				

#Ethnoveterinary medicinal uses

Recorded data were analyzed quantitatively with the help of 8 suitable statistical tools and the results were presented in Tables 1 and 2. From the quantitative analyses of the collected data, it has been observed that the value of ethnobotanical richness (ER) of *P*. *tuberosa* is 0.76, and the UKI value of this plant species is 1.12, which indicates the in-depth knowledge base regarding uses of the plant among the knowledgeable persons of the society. KVI calculated for this plant was 51.35, which illustrates the high knowledge level of the informants regarding the use of *P*. *tuberosa*.

**Table 2:** Determination of ethnobotanical richness (ER), use knowledge index (UKI), knowledge value index (KVI), ethnomedicinal income index (EI), and tissue importance value (TIV) of *P*. *tuberosa*.

Parameters	Total number
Total number of persons interviewed	185
Persons use the plants for healing purposes	125
Respondents not interested in herbal medicine	60
Number of informants familiar with the uses of *P*. *tuberosa*	95
Persons generate income by marketing tuber of *P*. *tuberosa*	20
Respondents who know the plant in their local/tribal name	85
**Indices used**	**Values**
Ethnobotanical richness (ER)	95/125 = 0.76
Use knowledge index (UKI)	95/85 = 1.12
Knowledge value index (KVI)	95/185 = 51.35
Ethnomedicinal income index(EI)	20/95 = 21.05
Tissue importance value (TIV):	
i) TIV_(Tuber)_	95/95 = 1
ii) TIV_(Leaf)_	15/95 = 0.16
iii) TIV_(Seed)_	15/95 = 0.16

The plant *P*. *tuberosa* is called by different local names and the name ‘Bhuin-kumro’ mentioned by most of the informants does indicate/ highlight the morphological similarity of its rounded swollen tuber with a pumpkin. The name Bhuin-kumro earned the highest score of NHI (82.35) among the local names recorded (S1 Table). In most of the cases, tuber of this plant is collected from the wild. Among the interviewees, only 20 informants occasionally marketed the tuber for gaining of some financial advantage; thisfinding led to a low EI value of the studied plant (EI = 21.05). Uses of 3 plant parts, namely leaves, seeds, and tubers, were recorded in the present investigation. The tissue importance value of the plant parts was found to be maximum for the tuber of *P*. *tuberosa* (TIV = 01) because 76% of the interviewees (95 informants) were very much aware about the usefulness of the studied plant part and were able to recall at least one use of it. The tuber part of this plant had also been considered as one of the vital plant parts used for mainly medicinal purposes since the Vedic age. Scientific studies made in the recent past reveal a varied range of health benefits of the tuber which is reasonably correlated with the presence of many bioactive compounds like puerarin, daidzin, genistin, puerarone, tuberosin and others explored through phytochemical investigation [11].

The use-value (UV) of the plant species ranges from 0.08 to 0.52, and the fidelity level (FL) score exhibits a range from 10.53 to 68.42%. It has been noticed that against helminth infection of veterinary animals, scores of these two indices namely, UV (0.52) and FL (68.42%), were attractively higher among the recorded uses of the concerned plant, which clearly highlight its ethnomedicinal reliability as a herbal anthelmintic agent.

After the analysis of both qualitative and quantitative ethnomedicinal data few interesting facts came into the spotlight: (i) ethnomedicinal use of *P*. *tuberosa* tuber in treatment of helminthiasis of veterinary animals is reported here for the first time from eastern India, (ii) the tissue importance value attributed to the tuber of *P*. *tuberosa* is very high compared to other parts of the plant which addresses the chemical as well as pharmacological sifnificance of this variously used plant part, and (iii) the reliability of *Pueraria* tuber against helminthiasis is strengthened by the high scores of FL and UV estimated for this purpose.

All these ethnomedicinal facts converse into an ethno-guided lead which illustrates the convincing information regarding traditional uses of root tuber of this plant as an effective anthelmintic agent. Considering this ethno-guided information, phytochemical profile, anthelmintic property and toxicity threats of the tuber part of *P*. *tuberosa* were studied.

### 3.2. Phytochemical analyses

#### 3.2.1. Estimation of total phenol, total flavonoid, total tannin, and total alkaloid

**Fig 3:**
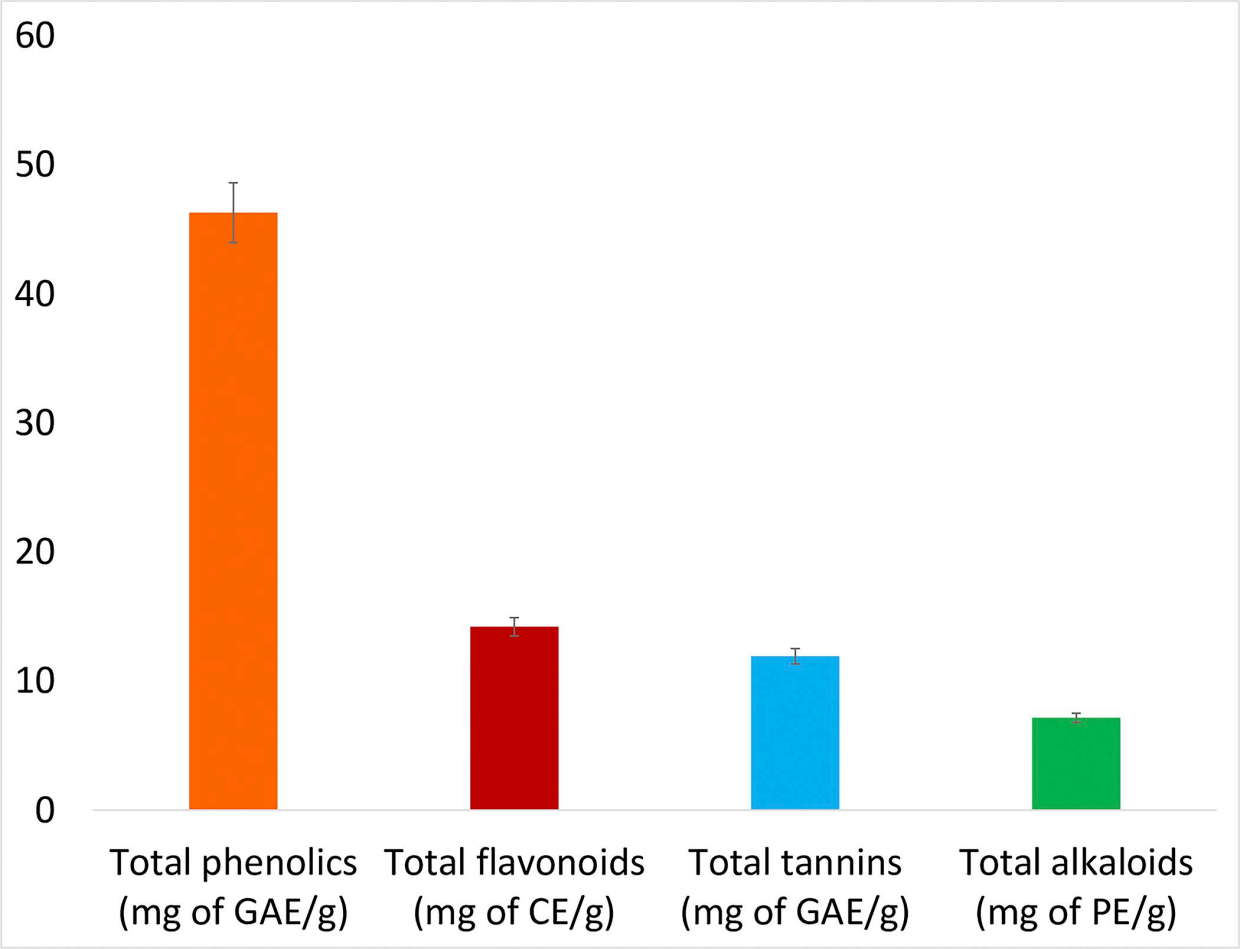
Phytochemical profiles of methanolic tuber extract of *P*. *tuberosa*.

Phytochemical screening has revealed that the tuber of *P*. *tuberosa* contains a considerable amount of medicinally important phytochemicals like phenolics, flavonoids, tannins and alkaloids (46.29 mg of GAE/gm, 14.2 mg of CE/gm, 11.92 mg of GAE/gm and 7.15 mg of PE/gm, respectively) (Fig 3). Numerous researches have established that these groups of phytochemicals play a very potent role while curing different health disorders, including helminthiasis [47, 53, 54]. Phenolic compounds such as ellagic, gentisic and gallic acids from *Anogeissus leiocarpus* were studied successfully for their anthelmintic potentiality on the helminth models like *Onchocerca ochengi* and *Caenorhabditis elegans* [55]. Tannins are the widely studied phytochemical group for their anthelmintic property in small ruminants. Iqbal et al. have studied the condensed tannins for their direct and indirect anthelmintic efficiencies and found them beneficial in reducing the hatching rate of nematode eggs in sheep [56]. The tannins have manifested their ability to change the physical and chemical properties of protein molecules present in the cuticle layer, esophagus, oral cavity, vulva and cloaca of the nematodes [57, 58]. Flavonoid compounds present in different plants like *Turnera ulmifolia*, *Digitaria insularis*, *Agave sisalana* have already been evaluated for their anthelmintic potentialities and found effective in reducing the hatching of eggs, larval development and its survivability [59–61]. In a recent study, it has been noticed that pods of *Acacia farnesiana*, a leguminous plant, contain nematocidal flavonoid compounds which are very much effective against *Haemonchus contortus* [62]. Alkaloids are another group of phytochemicals that have immense medicinal importance [63]. Several studies have shown the efficiency of alkaloids in controlling endoparasitic infections [64–67].

Chemical investigation of *P*.*tuberosa* highlights the presence of certain phytochemical groups of proven anthelmintic properties in its tuber part and such observation clearly indicates the rationale of uses of this plant as an anthelmintic veterinary drug in folk-medicine.

#### 3.2.2. HPLC analyses

For HPLC analysis, a total of five standards of phenol and flavonoid compounds were used. After the qualitative and quantitative estimation of these therapeutically important phytochemicals from the aqueous, ethanol and methanol extracts of *P*. *tuberosa* tuber, it was noticed that Catechin was present in considerable amount in all the solvent extracts; maximum in methanolic extract (629.928 μg/mL) followed by ethanolic extract (145.662 μg/mL), and aqueous extract (5.651 μg/mL). The other phenolic compounds estimated in the highest quantities from different solvent extracts are Gallic acid (5.036 μg/mL in ethanol extract), Naringin (3.6 μg/mL in ethanol extract), and Quercetin (1.030 μg/mL in methanol extract). Detailed result of HPLC study has been provided in (S1–S4 Tables; S1 Fig). All these phenolic and flavonoid compounds detected in various solvent extracts of *P*. *tuberosa* have already been established with experimental evidences as effective anthelmintic agent [47, 68, 69]. Presence of the substantial amount of Catechin in the methanol extract is a good indication of the anthelmintic potentiality of the investigated plant. This fact could be justified by earlier workers on the role of Catechin to control helminth parasites [70, 71].

#### 3.2.3. GC-MS analyses

From the GC-MS analyses, a total of 12 peaks have been identified by comparing their peak area (%), height (%), peak retention time, and mass spectral fragmentation patterns to that of the known compounds documented in the National Institute of Standards and Technology (NIST) library (S2A Fig). A total of 12 compounds have been identified,. Most of these compounds are biologically active and show a wide range of medicinal efficacy (Table 3; S2B Fig).

**Table 3:** List of the chemical compounds identified from methanolic extract of *P*. *tuberosa* tuber by GC-MS analysis and their reported biological activity.

Sl. No.	Referred number of NIST and CAS*	Chemical compound and (Molecular formula)	*t*_R_ (minute)**	Mol. weight	RA (%)***	Reported Bioactivity	References
1.	NIST: 36550 CAS: 14852-31-4	2-Hexadecanol (C_16_H_34_O)	3.808	242	7.875	Antibacterial activity against *Propionibacterium acnes*	[123]
2.	NIST: 111636 CAS: 959100-16-4	2-Myristynoyl pantetheine (C_25_H_44_N_2_O_5_S)	17.1	484	4.655	Antimicrobial activity	[124]
3.	NIST: 16824 CAS: 6980-45-6	4aα, 4bβ-Gibbane-1α, 10β-dicarboxylic acid, 4a-formyl-7-hydroxy-1-methyl-8-methylene-, dimethyl ester (C_22_H_30_O_6_)	13.764	390	6.208	It is Gibberellin GA24, methyl ester	Unknown
4.	NIST: 36057 CAS: 17364-34-0	6,9,12,15-Docosatetraenoic acid, methyl ester (C_23_H_38_O_2_)	9.89	346	10.396	Cardioprotective, hypocholesterolemic	[122]
5.	NIST: 28309 CAS: 40013-87-4	9,10-Secocholesta-5,7,10(19)-triene-3,24,25-triol, (3β, 5Z,7E)- (C_27_H_44_O_3_)	13.896	416	7.085	Antifungal and antibacterial activities, Antiviral, anti-Parkinsonism	[117, 119]
6.	NIST: 43703 CAS: 2091-29-4	9-Hexadecenoic acid (C_16_H_30_O_2_)	6.148	254	9.114	Antifungal activity, Anti-inflammatory activity, Antieczematic,	[125–127]
7.	NIST: 36520 CAS: 56599-46-3	9-Octadecenoic acid, (2-phenyl-1,3-dioxolan-4-yl)methyl ester, trans- (C_28_H_44_O_4_)	16.785	444	27.691	Nematicide, anti-arthritic, anti-acne, hepatoprotective, anti-androgenic, anti-histaminic, anti-inflammatory	[118]
8.	NIST: 245762 CAS: 6386-38-5	Benzenepropanoic acid, 3,5-bis(1,1-dimethyl)-4-hydroxy-, methyl ester (C_18_H_28_O_3_)	6.56	292	4.833	Antifungal and antioxidant property	[120]
9.	NIST: 35855 CAS: 10152-65-5	Cyclopropanedodecanoic acid, 2-octyl-, methyl ester (C_24_H_46_O_2_)	13.396	366	7.081	Anticancer, antitumor, antiestrogenic, antimicrobateria	[121]
10.	NIST: 43053 CAS: N/A	Ethyl iso-allocholate (C_26_H_44_O_5_)	16.471	436	6.207	Anti-inflammatory activity, antimicrobial activity	[116, 117]
11.	NIST: 129096 CAS: N/A	Morphinan-4,5-epoxy-3,6-di-ol,6-[7-nitrobenzofurazan-4-yl]amino- (C_26_H_27_N_5_O_6_)	25.174	505	6.853	Unknown	Unknown
12.	NIST: 48960 CAS: 58072-54-1	Spirost-8-en-11-one, 3-hydroxy-, (3β, 5α, 14β, 20β, 22β, 25R)- (C_27_H_40_O_4_)	13.947	428	6.808	Antipyretic and antiinflammatory	[119]

*Chemical abstract service (CAS), National Institute of Standards and Technology (NIST)

**Retention time (*t*_R_)

***Relative abundance (RA)

### 3.3. Role of proximate and mineral content

Livestock infested with gastrointestinal parasites symptomized by a progressive decline in feed intake, impaired nutrient utilization, loss in body weight, diarrhoea, malnutrition, internal tissue damage, anaemia, and sometimes death may occur [72, 73]. During the time of parasite infestation, nutrient intake is often compromised and available nutrients are utilized by the diseased animals for high survival priority [74]. So, nutritional deficiencies commonly occur as a consequence of such a disease. Deficiency in protein and over all calorie requirement is commonly evident in the dietary intake in this case. Not surprisingly, deficiencies of mineral nutrients like Na, K, Mg, Zn, and Ca are also common and should be supplemented as soon as possible [75].

Proximate analysis showed (Table 4) that the mature root tuber of *P*. *tuberosa* contains a high amount of carbohydrate (32.62 ± 0.87 gm/100gm of dry weight), protein (10.69 ± 0.37 gm/100gm of dry weight), and crude fiber (29.12 ± 1.04 gm/100gm of dry weight). The tuber part is characterized by high caloric value, i.e.197.659 ± 3.73 Kcal/100gm of tissue.

**Table 4:** Proximate and mineral contents of *P*. *tuberosa* tuber.

Parameters	Amount
**Na (**mg/100 gm**)**	4.4 ± 1.67
**K (**mg/100 gm**)**	97 ± 9.33
**Ca(**mg/100 gm**)**	242 ± 13
**Mg (**mg/100 gm**)**	105 ± 9.87
**Zn (**mg/100 gm**)**	5.2 ± 1.58
**Mn(**mg/100 gm**)**	1.49 ± 0.77
**Fe (**mg/100 gm**)**	12.2 ± 2.77
**Fat content (**gm/100 gm**)**	2.16 ± 0.05
**Crude Protein content (**gm/100 gm**)**	10.69 ± 0.37
**Carbohydrate (**gm/100 gm**)**	32.62 ± 0.87
**Crude fibre (**gm/100 gm**)**	29.12 ± 1.04
**Caloric value(**Kcal/100 gm**)**	197.659± 3.73
**Moisture content (%)**	12.53±0.31
**Ash content (%):**	11.38± 0.19
**Acid insoluble (%):**	1.64± 0.08
**Water soluble (%):**	23.51± 1.27

Mineral content analysis illustrated (Table 4) that 100 gm of the dry tissue contain considerable amounts of essential minerals like Ca (242 ± 13 mg), Mg (105 ± 9.87 mg), K (97 ± 9.33 mg), Fe (12.2 ± 2.77 mg) and Zn (5.2 ± 1.58 mg).

There are substantial shreds of evidence regarding the importance of protein and minerals to control endoparasitic infection in livestock [76, 77]. Infection of endoparasites causes a significant decline in several hematobiochemical parameters of host livestock. Some researchers have noticed that increased protein supply can improve the hematobiochemical parameters like albumin, total plasma protein, packed cell volume (PCV) and immunoglobulin (IgA) of a host animal [78, 79]. Apart from protein, livestock requires some essential minerals for its proper growth and building the immunity [80]. Among the minerals, copper, iron and zinc have been studied for their effective role in host resistance against endoparasitic infection [81]. Nowadays, copper supplement is given to the livestock to control the endoparasitic diseases with long-term effect [77]. Though iron has no direct impact on preventing the endoparasites but it helps in increasing the level of hematocrit and hemoglobin when given to the infected animal as a mineral supplement [82]. Zinc is an established co-factor of several enzymes and thus responsible for many biochemical processes that boost up the immune response of the host animals which markedly decline the rate of endoparasitic infection [83]. The presence of a considerable amounts of protein, zinc and iron in the studied plant strengthens the idea that it may have some definite role while preventing and curing the helminthiasis of livestock.

Apart from their supportive role in curing helminthiasis, proximate and mineral content of *P*. *tuberosa* can provide dietary support to promote health in livestock. High carbohydrate content of *P*. *tuberosa* can supply majority of the energy required for the development, production and growth of livestock [84]. For the growth and repair of tissues as well as the development of muscles, animals need protein like essential macronutrient [85]. For large ruminants like dairy cows, crude fiber is an essential feed component which not only supplies energy and nutritional benefits but also acts as a regulatory factor for maintenance of gut health and feed intake [86]. Likewise, the studied mineral nutrients like Ca, Mg, K, Fe, and Zn are also essential in livestock for milk production, immune system activation, energy metabolism, hormone and vitamin synthesis, blood cell formation, enzyme regulation, and formation of skeletal system [87]. So, all these essential macro and micronutrients present in adequate quantities in the root tuber of studied plant can possibly help to maintain good health in livestock when given a considerable amount as animal feed.

Proximate and mineral content analysis of *P*. *tuberosa* indicates the nutritional richness of its root tuber, which can promote sufficient health benefits to the livestock and plays a vital supportive role in developing resistance against the intestinal parasites.

### 3.4. Pharmacological activity

#### 3.4.1. Antioxidant activity

ABTS and DPPH assays have revealed that the methanolic solvent extract of *P*. *tuberosa* tuber exhibits promising antioxidant potentials with the lowest IC_50_ values (86.29 μg/mL in DPPH and 75.63 μg/mL in ABTS assay). The results of TAC assay manifested a similar trend as observed in the previous two assays (Table 5).

**Table 5:** Antioxidant potential of different solvent extracts of the *P*. *tuberosa* tuber.

Antioxidant activity	Different solvent extracts		
	*Water*	*Methanol*	*Ethanol*
DPPH radical scavenging activity (IC_50_ value in μg/mL)	106.52 ± 0.59	86.29 ± 0.65	90.25 ± 0.29
ABTS radical scavenging activity (IC_50_ value in μg/mL)	100.74 ± 0.25	75.63 ± 0.71	69.33 ± 0.58
Total antioxidant capacity (mg ascorbic acid equivalent or, mg AAE/gm)	29.33 ± 0.39	41.33 ± 0.43	47.04 ± 0.25

Several studies have reported that the level of reactive oxygen species (ROS) is increased in the parasites infected host cells, thus host tissue faces more oxidative stress and its harmful consequences [88, 89]. Antioxidant molecules play a pivotal role in scavenging such free radicles accumulated in the cells and check the cell as well as tissue damage [90]. Different *in vitro* and *in vivo* studies evaluated the antioxidant capacity of *P*. *tuberosa* and reported effective inhibition of lipid peroxidation with potent superoxide and hydroxyl radical scavenging activities [91, 92]. In a recent study, authors have tested the novel therapeutic approach based on a combination of anthelmintic drugs with antioxidant biomolecules against a helminth parasite, *Schistosoma mansoni* and a marked synergistic effect of the antioxidant molecules on activity of the standard anthelmintic drugs (praziquantel and artesunate) was observed [93].

Several antioxidant compounds such as catechin, gallic acid and quercetin, in substantial amounts are evident in various extracts of the *P*. *tuberosa* tuber during our HPLC study. The presence of these bioactive compounds in the tuber of this tested plant provides the opportunity to explore their synergistic effect thoroughly while combating helminth parasites of livestock, as previous studies have justified their potentiality as anthelminthic agents [69, 70, 94].

#### 3.4.2. Anthelmintic activity

*3*.*4*.*2*.*1*. *Effectiveness against trematode Cotylophoron cotylophorum*. Paramphistomosis (stomach fluke disease) in small ruminants is caused by digenetic trematodes and among them, the most prevalent one is *Cotylophoron cotylophorum*. Both the disease and the parasite are widely prevalent in India and exhibit most harmful effect during and after the monsoon [95] leading to severe morbidity and mortality of the host animals [96]. Aqueous, ethanolic and methanolic extracts of *P*. *tuberosa* was evaluated against two eco-morphologically significant forms of *C*. *cotylophorum* at various concentrations and affectivity was monitored by MTT assay.

**Fig 4:**
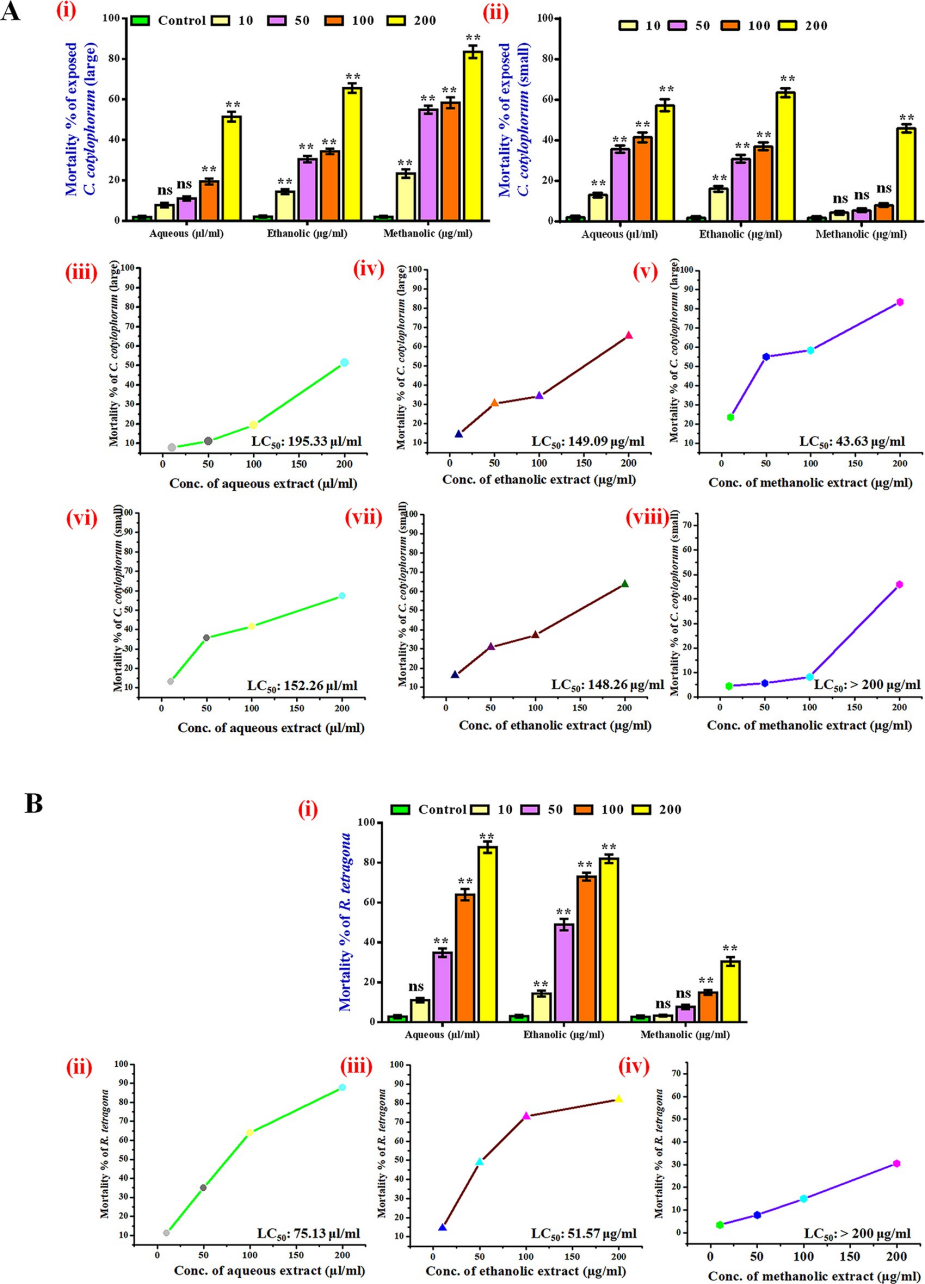
**(A)** Estimation of anti-trematode activity of aqueous, ethanolic and methanolic extracts of *P*. *tuberosa* against *C*. *cotylophorum*. (i) Mortality of large and (ii) small *C*. *cotylophorum* in MTT assay. Evaluation of LC_50_ values against the large *C*. *cotylophorum* after exposure to aqueous (iii), ethanolic (iv) and methanolic (v) extracts. LC_50_ values were also evaluated against the small *C*. *cotylophorum* trematode separately after treatment with aqueous (vi), ethanolic (vii) and methanolic (viii) extracts. All values are expressed as mean ± SEM from triplicate assays from three independent experiments [P values ≤ 0.05 (*) or ≤ 0.01 (**)] vs. control of the respective treatment group. **(B)** Determination of the effectiveness against chicken cestode *R*. *tetragona*. (i) Mortality of the cestode *R*. *tetragona* was evaluated after 24 hof exposure to different extracts of *P*. *tuberosa* tuber by MTT assay. Evaluation of LC_50_ values against the cestode after exposure to aqueous (ii), ethanolic (iii) and methanolic (iv) extracts. All values are expressed as mean ± SEM from triplicate assays from three independent experiments [P values ≤ 0.05 (*) or ≤ 0.01 (**)] vs. control of the respective treatment group.

After 24 hours of exposure, the mortality rate of small and large variant of *C*. *cotylophorum* was evaluated. The maximum mortality was 51.5% when exposed to 200μL/mL of aqueous extracts (Fig 4Ai). Treatment with aqueous extracts demonstrated increasing mortality with increasing dosages (Fig 4Ai). When *C*. *cotylophorum* large trematodes were treated with ethanolic and methanolic extracts at 200 μg/mL of each, mortality was shown to be greater and was determined to be 65.7% and 83.6%, respectively (Fig 4Ai). In case of small trematode mortality of *C*. *cotylophorum* was also found significant (P value ≤ 0.01) after treatment with the different extracts (Fig 4Aii). Against the small trematodes, the highest mortality was noticed when exposed to ethanolic extract of 200μg/mL and found to be 63.6%. Against the aqueous (200μl/mL) and methanolic (200μg/mL) extracts the mortality was 57.4% and 45.9% respectively (Fig 4Aii). The difference in mortality of the two different forms signifies their selective absorption as well as susceptibility to different extracts. The specific vulnerability of the different forms of the same species possibly depends on their selective trophic level preference inside the host rumen, the normal residential site. Determination of LC_50_ value against any pathogen, when exposed to chemo-prophylactic measures, is very important in drug development research as it provides a critical perspective of their effectiveness and application potential [97]. LC_50_ values of different extracts against large and small trematodes of *C*. *cotylophorum* were evaluated (Fig 4Aiii–4Aviii). It was noticed that against the larger forms, the extracts showed better effectiveness as depicted by lower LC_50_ values compared to the LC_50_ values against the smaller *C*. *cotylophorum* trematode. Among all the extracts, methanolic extract showed the lowest LC_50_ against the large *C*. *cotylophorum* trematode and which is 43.63μg/mL (Fig 4Av). Ethanolic extract exhibited almost equal LC_50_ values against both the forms of the trematode parasite, which were 149.1μg/mL and 148.3μg/mL respectively against the large and small forms of the trematode (Fig 4Aiv and 4Avii).

**Fig 5:**
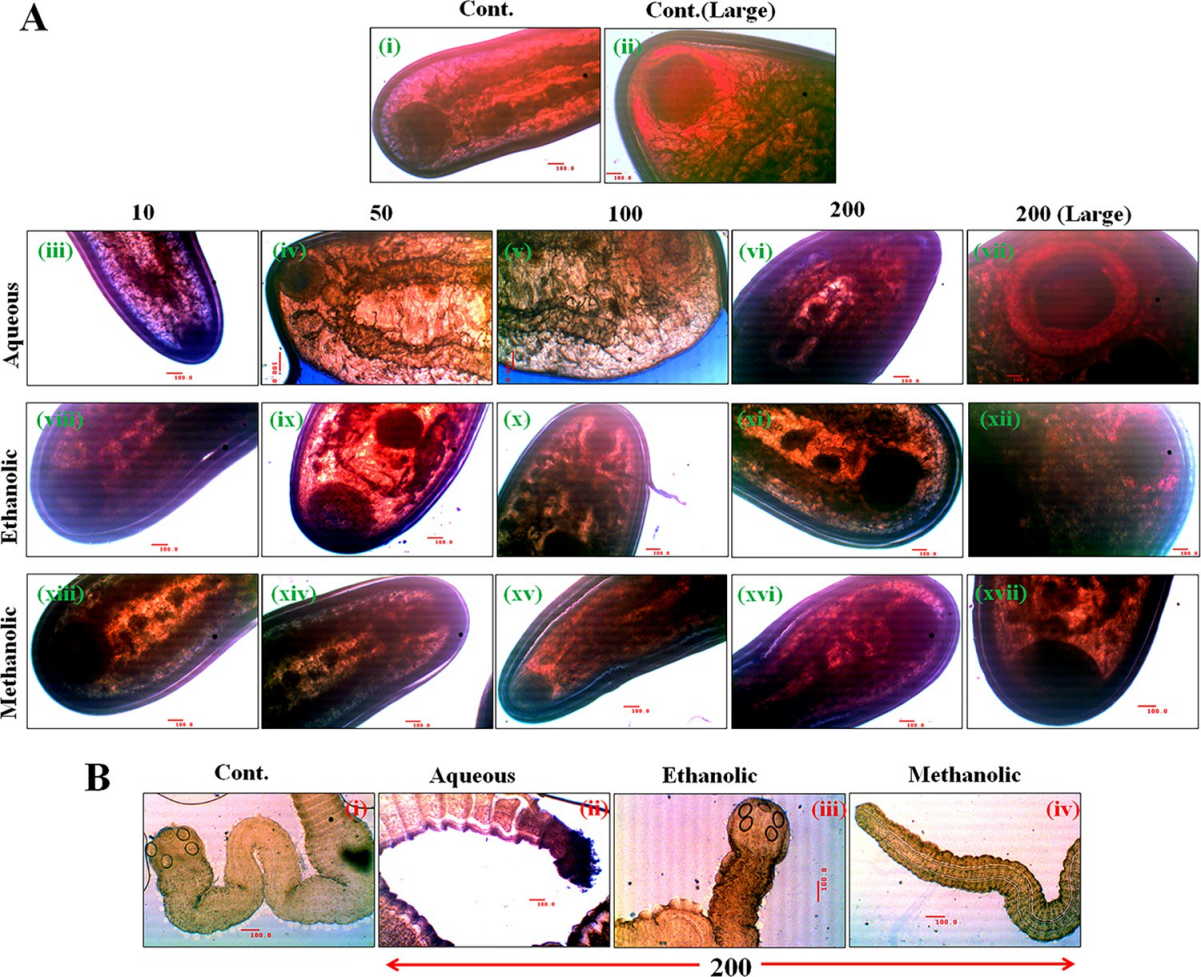
Evaluation of morphological aberration and confirmation of the presence of dead cells by Trypan blue staining of the aqueous, ethanolic and methanolic extracts treated *C*. *cotylophorum* trematode **(A)** and *R*. *tetragona* cestode **(B)**. Images are the representative of three separate experiments.

Control and different extract exposed parasites when micrographed under an inverted bright field microscope, control parasites (Fig 5Ai and 5Aii) were appeared as mostly red to pink, besides multiple blue patches were observed inside the treated parasites which indicates the presence of plenty of dead cells within it. From the MTT results and micrographs, it became apparent that *P*. *tuberosa* extracts were potent enough to kill the parasites *in vitro* and denoted their effectiveness, however, with a variable amplitude dependent upon the extraction procedure and application of doses.

*3*.*4*.*2*.*2*. *Effectiveness against cestode Raillietina tetragona*. Aqueous and ethanolic extracts were found effective against the parasite within the applied dose range (Fig 4B) where mortality was noticed to be 87.8% and 82%, respectively at the maximum dose of 200μg/mL (Fig 4Bi). At the lower doses of these two extracts the mortality was also considerably high. However, it was found that the methanolic extract was somewhat relatively ineffective in eliminating large numbers of cestode because mortality rate increased upto ~30.5% when exposed to highest concentration like 200μg/mL of it (Fig 4Bi). LC_50_ values of the extracts were calculated against *R*. *tetragona* after 24 h of exposure (Fig 4Bii–4Biv). Ethanolic extract showed the minimum LC_50_ value and it was 51.57μg/mL (Fig 4Biii) followed by aqueous extract’s value of 75.13μg/mL (Fig 4Bii). However, LC_50_ value of methanolic extract was beyond 200μg/mL and thus it was not possible to ascertain the exact value.

The importance was given to the scolex or head region of *R*. *tetragona* when micrographed(Fig 5Bi–5Biv). As opposed to the control worms (Fig 5Bi), which were mostly translucent and brownish-yellow in appearance, the presence of dead cells stained in blue was apparent in the parasites exposed to aqueous and ethanolic extracts, where as moderately less number of dead cells were noticed in methanolic extract exposed parasite (Fig 5Bii–5Biv). Blue stained dead cells were visible in highest number in the aqueous extract treated cestodes which stand for its enhanced effectivity against *R*. *tetragona* among the solvent extracts tested.

Domestic chickens (*Gallus gallus domesticus*) are one of the economically important poultry bird in rural India and substantial economic loss can happen if infected by cestode parasite like *Raillietina* sp. which can cause growth alteration, weakness, obstructive bowel movement in the chikens [98–100]. Present finding can be a great hope for the marginaliged rural people.

**Fig 6:**
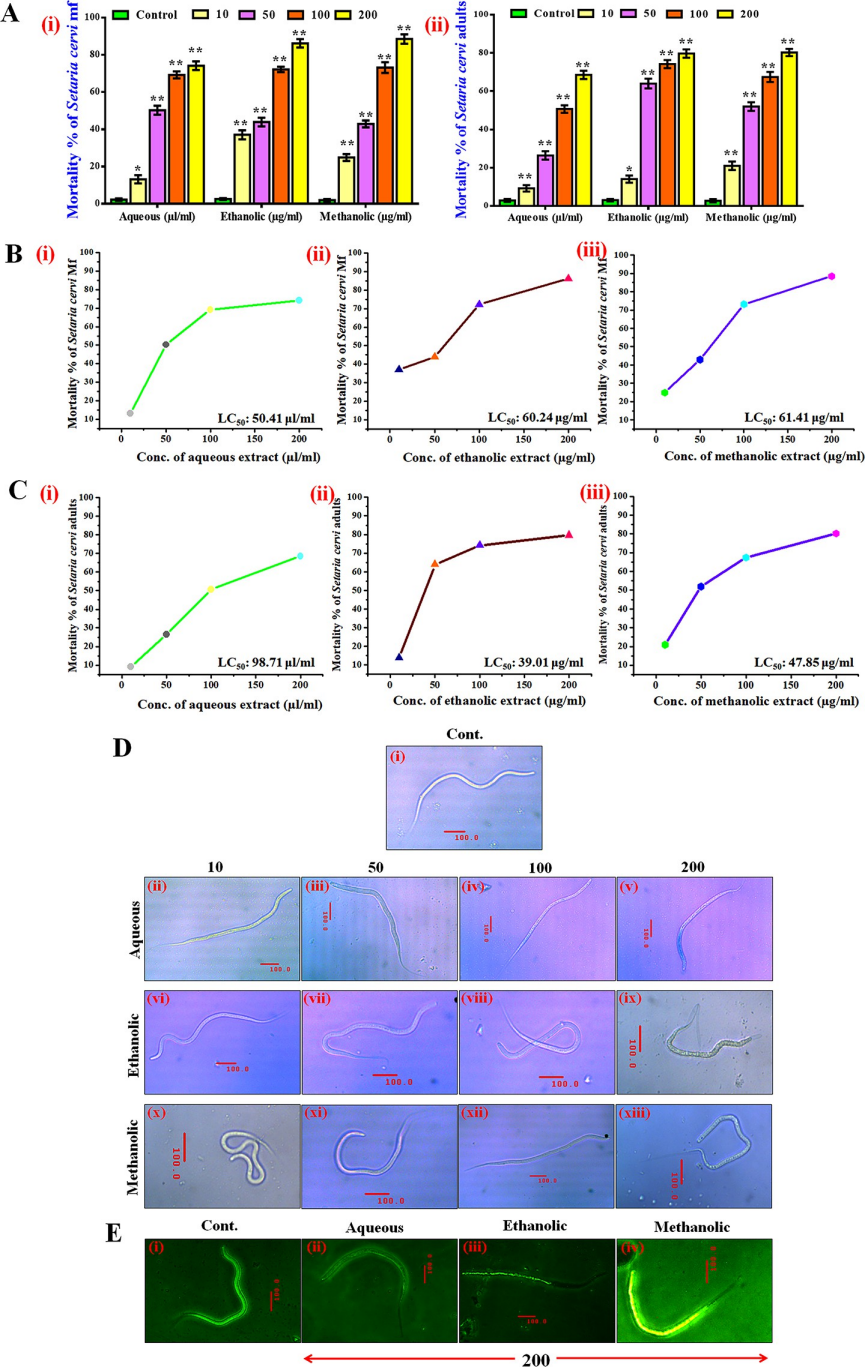
Determination of the effectiveness against bovine filarial nematode *S*. *cervi*. **(A)** Mortality of the mf (i) and adults (ii) of *S*. *cervi*was evaluated after 24 h of exposure to different extracts of *P*. *tuberosa* tuber by MTT assay. **(B)** Evaluation of LC_50_ values against the mf of *S*. *cervi* after exposure to aqueous (i), ethanolic (ii) and methanolic (iii) extracts. **(C)** Evaluation of LC_50_ values against the *S*. *cervi* adults after exposure to aqueous (i), ethanolic (ii) and methanolic (iii) extracts. All values are expressed as mean ± SEM of three independent experiments [(P values ≤ 0.05 (*) or ≤ 0.01 (**)] vs. control of the respective treatment group. **(D)** Evaluation of morphological aberration and confirmation of the presence of dead cells by Trypan blue staining of the exposed *S*. *cervi* mf. **(E)** Phase-contrast micrographs of exposed *S*. *cervi* mf. Images are representative of three separate experiments.

*3*.*4*.*2*.*3*. *Antifilarial activity against nematode Setaria cervi*. Chemoprophylactic measure against the adult of filarial nematodes is precisely inadequate [101, 102] and thus a potential adulticide that can also act against the mf can be of high promise in the field of anti-filarial drug development research. A preliminary *in vitro* MTT assay has shown that all the three extracts i.e., aqueous, ethanol and methanol were highly effective on the % mortality of mf stages of *S*. *cervi* (Fig 6Ai). The highest mortality (88.5%) was noticed when mf were exposed to the dose of 200μg/mL of methanolic extract and at the same dose, the mortality of mf was found to be 77.1% and 86.2% respectively for aqueous and ethanolic extracts (Fig 6Ai). LC_50_ values of the extracts against the mf stages of *S*. *cervi* was found variable in all the three extracts (Fig 6Bi–6Biii) and it was minimum for the aqueous extract with LC_50_ value of 50.41μg/mL and for the ethanolic and methanolic extracts, the LC_50_ values were 60.24μg/mL and 61.41μg/mL respectively. Against the adults of the *S*. *cervi* parasite, the mortality trend was almost similar to that of mortality observed in mf stage of the parasite. The highest mortality was noticed when the adults were exposed to a methanolic extract of 200μg/mL and the mortality rate was noticed to be 80.2% (Fig 6Aii). Against the aqueous and ethanolic extracts, the adult mortality was found to be 68.6% and 79.7%, respectively (Fig 6Aii). The lowest LC_50_ value against the adult parasites was noticed for the ethanolic extract and the value was 39.01μg/mL (Fig 6Cii).

The most prominent alteration of morphology and the highest percentage of dead cells were noticed in mf exposed to the concentration of 200μg/mL of ethanolic extract (Fig 6Dix). It is also visible in case of the phase-contrast photomicrographs (Fig 6Eiii) where the parasite has lost almost its morphological integrity and has shrivelled inside its sheath. Thus from the MTT assay and the photomicrographs produced as supporting documents, it is evident that *P*. *tuberosa* tuber extracts were adequately effective against the filarial nematodes of animal origin.

### 3.5. *In silico* studies

The GC-MS study explored the presence of 12 phytocompounds in the tuber drug of *P*. *tuberosa*. The *in silico* studies have been carried out for these 12 phytocompounds to analyze their therapeutic potencies against sis target proteins selected from anti-inflammatory, antioxidant and anthelminthic categories. Initially, docking studies were performed employing the iGEMDOCK software to determine the binding affinities of each of the twelve phytocompounds to the selected target proteins. Using the iGEMDOCK the binding interactions and conformations of each phytocompound with each target protein were predicted and ligand-protein complexes were rated on the basis of lowest energy and total binding energy values.

Thus, among the 12 phytocompounds, Morphinan-4,5-epoxy-3,6-di-ol,6-[7-nitrobenzofurazan-4-yl]amino- exhibited the best binding conformations with the lowest binding energy values while binding with three categories of target proteins namely anti-inflammatory (-97.9918 kcal/mol), antioxidant (-107.776 kcal/mol), and anthelmintic [e.g., Asparaginyl tRNA synthase (-131.45 kcal/mol), GABA-A receptor (-117.973 kcal/mol), Glutathione S-Transferase I (-116.313 kcal/mol) and glutamate-gated chloride channel (-83.3181 kcal/mol)] (Table 6). Findings of the present work are confirmed by the findings of earlier research which depicted that the lower is the binding energy score, the greater is the protein-ligand binding stability [103].

**Table 6:** Estimated binding energy values of 12 phytocompounds with six target proteins [one anti-inflammatory (Tnf-alpha), one anti-oxidant (Glutathione reductase), and four anthelmintic proteins (e.g., Asparaginyl tRNA synthase, GABA-A receptor, Glutathione S-Transferase I and glutamate-gated chloride channel)].

Name of the compounds	Target proteins					
	TNF-alpha (kcal/mol)	Glutathione Reductase (kcal/mol)	Asparaginyl tRNA synthase (kcal/mol)	GABA-A receptor (kcal/mol)	Glutathione S Transferase-I (kcal/mol)	Glutamate-gated chloride channel (kcal/mol)
2-Myristynoyl pantetheine	-93.7639	-99.8452	-120.188	-116.515	-103.138	-78.8904
Benzenepropanoic acid, 3,5-bis (1,1-dimethylethyl)-4-hydroxy,methyl ester	-75.0279	-86.1636	-97.8743	-90.0187	-77.7234	-57.6277
6,9,12,15-Docosatetraenoic acid, methyl ester	-80.3515	-91.329	-107.739	-102.837	-79.9185	-66.5737
2- Hexadecanol	-66.9597	-89.3487	-101.281	-84.4975	-75.428	-58.4019
Cyclopropanedodecanoic acid, 2-octyl,methyl ester	-84.2751	-96.3201	-90.7008	-103.562	-82.8732	-72.5011
9-Octadecenoic acid, (2-phenyl-1,3-dioxolan-4-yl) methyl ester, trans	-88.1404	-87.7631	-106.138	-106.308	-83.0287	-81.1364
Morphinan-4,5-epoxy-3,6-di-ol, 6-[7-nitrobenzofurazan-4-yl] amino	-97.9918	-107.776	-131.45	-117.973	-116.313	-83.3181
9,10-Secocholesta-5,7,10(19)-triene-3,24,25-triol,(3β,5Z,7E)	-92.7895	-90.8563	-104.545	-98.0025	-97.7393	-78.4133
Ethyl iso-allocholate	-81.9327	-98.0994	-100.991	-114.856	-98.8275	-69.2404
Spirost-8-en-11-one, 3-hydroxy-,(3β,5α,14β,20β,22β,25R)	-76.3821	-106.977	-102.771	-108.81	-87.5739	-61.0841
4aα,4bβ-Gibbane-1α,10β-dicarboxylic acid,4a-formyl-7 -hydroxy-1-methyl-8-methylene-, dimethyl ester	-80.669	-96.7051	-113.648	-116.813	-91.0524	-64.4614
9-Hexadecenoic acid	-63.8326	-100.123	-99.2486	-84.0958	-82.6428	-53.358

**Fig 7:**
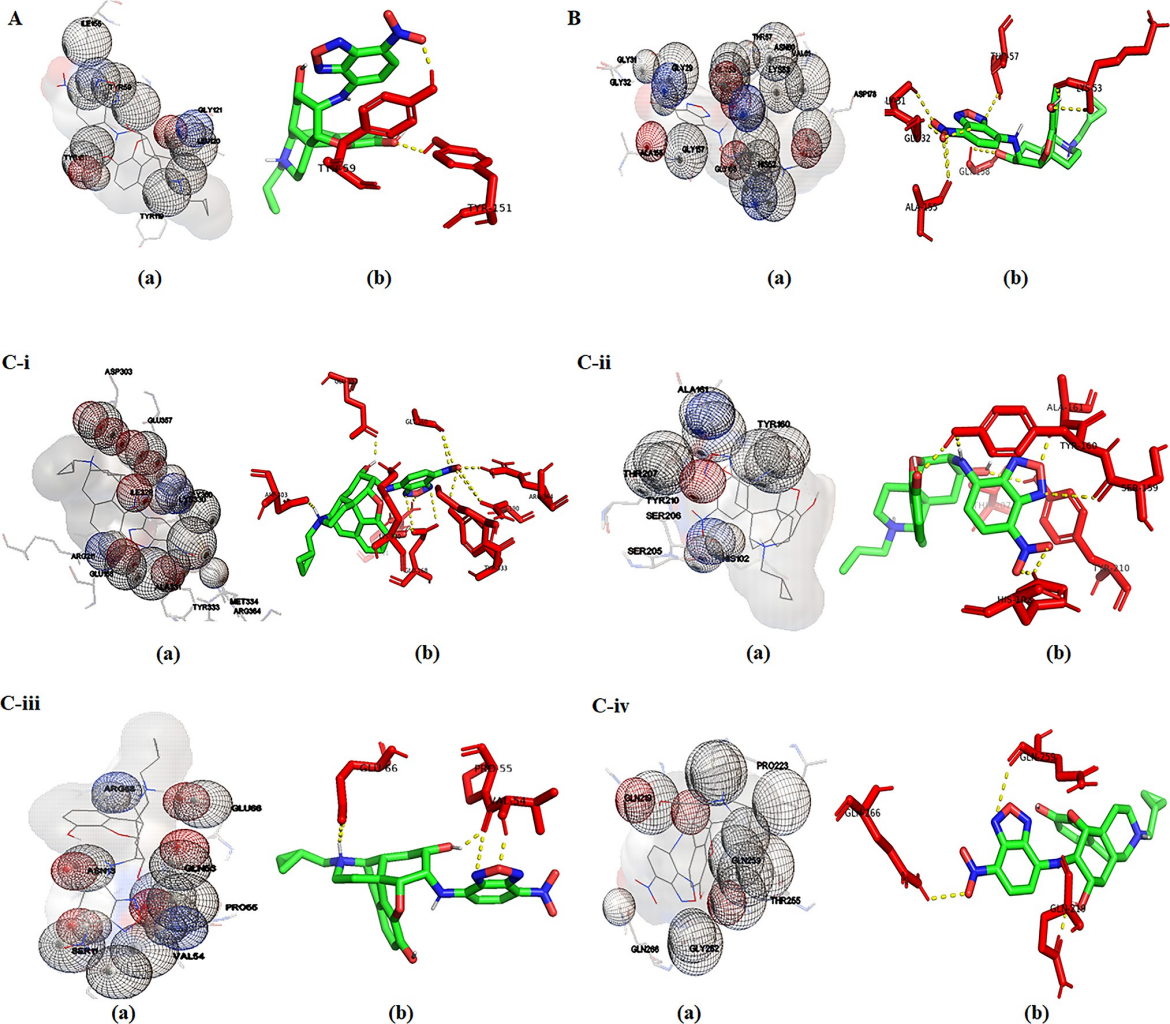
**A. (a)** Amino acid residues of the target protein TNF-α interacting with Morphinan-4,5-epoxy-3,6-di-ol, 6-[7-nitrobenzofurazan-4-yl]amino; **(b)** Visualization of the docked complex with pymol shows two hydrogen bond interactions (yellow dotted line) formed by the ligand involving two amino acid residues (Tyr151 and Tyr59) of target protein. **B: (a)** Amino acid residues of the target protein Glutathione Reductase interacting with Morphinan-4,5-epoxy-3,6-di-ol, 6-[7-nitrobenzofurazan-4-yl]amino; **(b)** Visualization of the docked complex with pymol shows nine hydrogen bond interactions (yellow dotted line) formed by the ligand involving six amino acid residues (Gly31.Gly32,Lys53,Thr57,Ala155 and Gly158) of target protein. **C-i: (a)**Amino acid residues of the target protein asparaginyl-tRNA synthetase interacting with Morphinan-4,5-epoxy-3,6-di-ol, 6-[7-nitrobenzofurazan-4-yl]amino; **(b)** Visualization of the docked complex with pymol shows twelve hydrogen bond interactions (yellow dotted line) formed by ligand involving eight amino acid residues (Glu168, Arg211, Asp303, Lys330, Tyr333, Glu357, Glu360, and Arg364) of target protein. **C-ii: (a)** Amino acid residues of the target protein GABA-A receptor interacting with Morphinan-4,5-epoxy-3,6-di-ol, 6-[7-nitrobenzofurazan-4-yl]amino; **(b)** Visulalization of the docked complex with pymol shows seven hydrogen bond interaction (yellow dotted line) formed by the ligand involving six amino acid residues (His102, Ser159, Tyr160, Ala161,Thr207 and Tyr210) of target protein.**C-iii: (a)** Amino acid residues of the target protein Glutathione S-transferase-I interacting with Morphinan-4,5-epoxy-3,6-di-ol, 6-[7-nitrobenzofurazan-4-yl]amino; **(b)** Visualization of the docked complex with pymol shows four hydrogen bond interactions (yellow dotted line) formed by the ligand involving three amino acid residues (Val54, Pro55 and Glu66) of target protein.**C-iv: (a)**Amino acid residues of the target protein glutamate-gated chloride channel interacting with Morphinan-4,5-epoxy-3,6-di-ol, 6-[7-nitrobenzofurazan-4-yl]amino; **(b)** Visualization of the docked complex with pymol shows three hydrogen bond interactions (yellow dotted line) formed by the ligand involving three amino acid residues (Gln219, Gln259 and Gln266) of target protein.

Then the subsequent docking analysis has been performed for the best-binding compound (Morphinan-4,5-epoxy-3,6-di-ol,6-[7-nitrobenzofurazan-4-yl]amino-) identified from the iGEMDOCK study. This study enables to search for the best binding pose of the phytocompound along with a series of energy values such as binding energy, ligand efficiency, and inhibition constant for each target protein (Table 7). The docking study revealed the potential strength of Morphinan-4,5-epoxy-3,6-di-ol,6-[7-nitrobenzofurazan-4-yl]amino- compound’s binding affinity into the binding sites of target proteins with minimum binding energy (ranging from -5.82 to -9.27 kcal/mol), ligand efficiency (ranging from -0.16 to -0.26 kcal/mol), inhibition constant (54.09. to 166.53 M). The compound also formed hydrogen-bond interactions and the best possible binding pose with the residues of anti-inflammatory (Fig 7A), antioxidant (Fig 7B), and anthelminthic (Fig 7Ci–7Civ) target proteins as shown by their corresponding 3D interaction models.

**Table 7:** Profile of the binding interactions of the compound Morphinan-4,5-epoxy-3,6-di-ol, 6-[7-nitrobenzofurazan-4-yl] amino with six target proteins using AutoDock 4.2 programme.

Name of the phytocompounds	Target proteins	Binding energy (ΔG) (Kcal/mol))	Ligand efficiency	Inhibition constant ((Ki) (μM))
Morphinan-4,5-epoxy-3,6-di-ol, 6-[7-nitrobenzofurazan-4-yl] amino	TNF-alpha	-5.82	-0.16	54.09
	Glutathione Reductase	-9.25	-0.25	166.53
	Asparaginyl tRNA synthase	-9.7	-0.26	77.77
	GABA-A receptor	-6.38	0.-17	21.08
	Glutathione S Transferase-I	-6.77	-0.18	10.9
	Glutamate-gated chloride channel	-6.27	-0.17	25.55

Molecular docking is now considered an efficient and cost-effective technique applied for designing and efficiency-testing of the drugs. This method offers information on drug-receptor interactions which can be used to anticipate how drug candidates will bind to their target proteins. The strong anthelminthic activity of tuber extracts of the investigated ethnomedicinal plant against three helmintic parasites (*C*. *cotylophorum*, *R*. *tetragona*, and *S*. *cervi*) prompted us to carry out the *in silico* for molecular docking studies of the 12 phytochemicals identified from its tuber extracts by GC-MS analysis to predict their possible mechanisms of action. Four anthelmintic proteins, namely asparaginyl tRNA synthase, GABA-A receptor, glutathione S-Transferase-I, and glutamate-gated chloride channel, were targeted for molecular docking studies along with one pro-inflammatory (TNF-α) and one antioxidant (Glutathione reductase) target protein. The compound Morphinan-4,5-epoxy-3,6-di-ol,6-[7-nitrobenzofurazan-4-yl]amino- was identified as the lead compound and it exhibited the best anti-inflammatory, antioxidant and anthelminthic activity among all twelve studied phytocompounds. Computational simulation studies revealed that Morphinan-4,5-epoxy-3,6-di-ol,6-[7-nitrobenzofurazan-4-yl]amino- showed a better affinity with the lowest binding energy than other compounds. The results of molecular docking studies also confirmed that various energy sources are consistent and they contribute to the overall strength of binding interactions of the compound for each target protein. Current findings of *in silico* analysis thus establish the compound Morphinan-4,5-epoxy-3,6-di-ol,6-[7-nitrobenzofurazan-4-yl]amino- very promising for developing an effective drug against helminth parasites. Further investigations to determine its bioactivity, pharmacokinetics, metabolism and other clinical properties are highly recommended for broad-spectrum drug discovery.

### 3.6. Cytotoxicity assay

The *in vitro* cytotoxicity study of hydromethanolic extract of *P*. *tuberosa* on the Vero cell line (ATCC- CCL-81) did not manifest any significant cell death even at its highest dose of 250 mg/L and morphology of the treated cells was also remained unaltered.

No statistically significant (P value ≤ 0.05) differences in body weights of the control and treated male and female mice have been observed here in the *in vivo* chronic toxicity test. The patterns of body weight gained by the treated mice were also very much comparable with the control groups and did not highlight any toxic effects. Apart from body weight, quality of fur, feeding behavior as well as activities remained unaltered throughout the treatment period. The hydromethanolic extract of the root tuber did not show any mortality or sign of toxicity in both males and females at the dose of 5000 mg/kg body weight over the observation period. Moreover, the treated mice did not exhibit any characteristic change in body weight during the experimental period. As no death of mice was observed in this experiment at the highest dose of administration, the LD_50_ of the methanolic extract could not be estimated and it was considered greater than 5000 mg/kg body weight, an experimental upper limit in the acute toxicity study. Thus, the high LD_50_ value is a strong pointer to the fact that the extract of this plant is considered safe.

Medicinal plant extracts, formulations, or the compounds derived from them have the advantage that they are frequently multi-targeted with no or insignificant toxic side effects. With recent emphasis of the WHO on the development of anthelmintic and antifilarial drugs from natural products, the current work was undertaken for screening of the extracts obtained from the tuber part of an ethnomedicinally diverse species *P*. *tuberosa*. From our ethnobotanical, phytochemical and pharmacological studies, it is understood that the tuber drug of *P*. *tuberosa* is a popularly used ethnomedicine with significantly high phenolics and other phytochemicals, considerably high antioxidant and anthelmintic activities. In the recent past, similar sorts of works have been carried out all over the globe to prove the traditional claims of certain medicinal plants while curing a wide range of diseases including helminthiasis [104–106]. Such scientific investigation of medicinal plants and their extracted products is necessary not only for exploring the novel natural products from it but also for examining the scientific basis of ethnomedicinal uses of those therapeutically potent plants. Despite its immense ethnomedicinal claims, as well as abundance of therapeutically significant phytochemicals profiled by HPLC and GC/MS analyses, systematic studies on toxicity of *P*. *tuberaosa* are indispensable to address the risks of toxicity and to determine its doses safe for livestock and human uses. Thus, to ensure the safety of this plant for livestock as well as human consumption, the hydromethanolic extracts of its tuber were tested for *in vitro* cytotoxicity and acute toxicity assays. In the present study, the plant’s tuber extracts had not shown any significant cell death against Vero cells (ATCC- CCL-81) using the MTT assay, even at the highest dose of 250 mg/mL. Subsequent to the *in vitro* cytotoxicity assay, the extract was tested on mice for acute toxicity study. Any behavioral abnormality as well as the death of any mice (including female and male) was not observed at a very high dose of 5000 mg/kg body weight. Thus, the use of this extract up to the highest dose was found entirely safe for the animals tested. According to the Harmonised system for classifying chemicals that cause acute toxicity adopted by the Organisation for Economic Co-operation and Development (OECD), this plant extract may be considered as a potent research material for further evaluating its non-toxic nature before recommended as safer drug for humans and other animals. So, further long-term toxicological and clinical investigations are highly recommended to confirm its safety and effectiveness in humans and livestock.

## 4. Conclusion

The present work on *P*. *tuberosa* (Roxb. ex Willd.) DC. provides some convincing evidences derived from five main areas of research such as ethnobotanical, phytochemical, bioactivity, toxicity and *in silico* studies and such findings could be instrumental for effective management of the endoparasitic diseases of livestock by formulating the cost-effective drugs from the active compounds responsible for anthelmintic potentiality of this plant.

The lucrative phytochemical profile of the *P*. *tuberosa* tuber is very much conviencing regarding its anthelmintic potentiality and different solvent extracts of the evaluated plant part showed effectiveness against all the experimental parasites such as *Cotylophoron cotylophorum*, *Raillietina tetragona* and *Setaria cervi*. Additionally, *in silico* molecular docking study revealed that the compound Morphinan-4,5-epoxy-3,6-di-ol, 6-[7-nitrobenzofurazan-4-yl] amino has significant potential as an anti-inflammatory and/or antihelminthic drug candidate, and this observation highlights the need for further isolation, characterization, *in vivo* studies and molecular mechanism of action of this novel phytocompound. Moreover, the nontoxic nature of the plant part will be worthwhile to the pharmacologists and clinicians to develop novel anthelmintic veterinary medicines.

## Supporting information

S1 Table Value of the name homogeneity index. (PDF)

S2 Table Quantification of selected phenolics and flavonoids from the aqueous extract of *P*. *tuberosa* tuber by HPLC analysis. (PDF)

S3 Table Quantification of selected phenolics and flavonoids from the methanolic extract of *P*. *tuberosa* tuber by HPLC analysis. (PDF)

S4 Table Quantification of selected phenolics and flavonoids from the ethanolic extract of *P*. *tuberosa* tuber by HPLC analysis. (PDF)

S1 Fig HPLC chromatogram of *P*. *tuberosa* tuber extracts: **(A)** Aqueous extract, **(B)** Methanolic extract, **(C)** Ethanolic extract.
(PDF)

S2 Fig **A.** GC-MS chromatogram obtained from methanolic extract of *P*. *tuberosa*; **B**. Chemical structures of the identified compounds.
(PDF)
