# Supplementary material for: An ethnopharmacological approach to evaluate antiparasitic and health-promoting abilities of Pueraria tuberosa (Willd.) DC. in livestock
Source: PLoS One. 2024 Jul 19;19(7):e0305667. doi: 10.1371/journal.pone.0305667 (PMC11259309; doi:10.1371/journal.pone.0305667)
Supplement: S1 Table — (PDF) [file pone.0305667.s001.pdf]

S1 Table : Value of the Name Homogeneity index (NHI)

Local/tribal name*	Number of informants know the plant by its name#	NHI value
**Bhuin-kumro (B/S)**	**70**	**82.35**
**Kanda palash (B)**	**15**	**17.65**
**Patal- kumro (B)**	**55**	**64.71**
**Patal-kondha (S)**	**30**	**35.29**
**Tirra (S)**	**05**	**0.06**

# Some informants know the plant by more than one name (B- Bengali; S- Santali)
