# Supplementary material for: An ethnopharmacological approach to evaluate antiparasitic and health-promoting abilities of Pueraria tuberosa (Willd.) DC. in livestock
Source: PLoS One. 2024 Jul 19;19(7):e0305667. doi: 10.1371/journal.pone.0305667 (PMC11259309; doi:10.1371/journal.pone.0305667)
Supplement: S2 Table — (PDF) [file pone.0305667.s002.pdf]

**S2 Table:** Quantification of selected phenolics and flavonoids from the aqueous extract of *P. tuberosa* tuber by HPLC analysis

| Peak No. | Ret.Time min | Peak Name | Height mAU | Area mAU*min | Ret. Area % | Amount µg/ml |
|---|---|---|---|---|---|---|
| 1 | 3.33 | n.a. | 229.854 | 64.747 | 23.91 | n.a. |
| 2 | 3.87 | n.a. | 76.357 | 28.438 | 10.50 | n.a. |
| 3 | 4.09 | n.a. | 11.456 | 0.767 | 0.28 | n.a. |
| 4 | 4.37 | n.a. | 75.789 | 19.338 | 7.14 | n.a. |
| 5 | 4.75 | n.a. | 71.358 | 10.505 | 3.88 | n.a. |
| 6 | 5.12 | n.a. | 6.687 | 1.000 | 0.37 | n.a. |
| 7 | 5.36 | n.a. | 82.038 | 8.779 | 3.24 | n.a. |
| 8 | 5.79 | GALLIC ACID | 7.561 | 0.835 | 0.31 | 0.735 |
| 9 | 6.04 | n.a. | 4.136 | 0.548 | 0.20 | n.a. |
| 10 | 7.90 | n.a. | 1.983 | 0.526 | 0.19 | n.a. |
| 11 | 10.02 | n.a. | 53.440 | 14.761 | 5.45 | n.a. |
| 12 | 10.59 | n.a. | 3.730 | 0.566 | 0.21 | n.a. |
| 13 | 11.36 | CATECHIN | 6.676 | 0.870 | 0.32 | 5.651 |
| 14 | 11.88 | n.a. | 26.178 | 4.304 | 1.59 | n.a. |
| 15 | 12.57 | n.a. | 64.753 | 20.091 | 7.42 | n.a. |
| 16 | 12.99 | n.a. | 5.124 | 0.760 | 0.28 | n.a. |
| 17 | 14.05 | n.a. | 54.872 | 21.790 | 8.05 | n.a. |
| 18 | 14.48 | n.a. | 6.213 | 0.846 | 0.31 | n.a. |
| 19 | 14.91 | n.a. | 4.745 | 0.718 | 0.27 | n.a. |
| 20 | 18.03 | n.a. | 71.266 | 18.224 | 6.73 | n.a. |
| 21 | 19.15 | n.a. | 4.469 | 1.486 | 0.55 | n.a. |
| 22 | 19.71 | n.a. | 5.028 | 0.876 | 0.32 | n.a. |
| 23 | 20.63 | NARINGIN | 2.933 | 0.542 | 0.20 | 0.755 |
| 24 | 21.35 | n.a. | 60.391 | 21.272 | 7.86 | n.a. |
| 25 | 22.67 | n.a. | 4.199 | 1.852 | 0.68 | n.a. |
| 26 | 30.09 | n.a. | 2.621 | 1.237 | 0.46 | n.a. |
| 27 | 31.62 | n.a. | 2.322 | 0.845 | 0.31 | n.a. |
| 28 | 41.53 | n.a. | 1.566 | 0.839 | 0.31 | n.a. |
| 29 | 42.76 | n.a. | 6.798 | 2.586 | 0.95 | n.a. |
| 30 | 46.55 | n.a. | 14.795 | 7.213 | 2.66 | n.a. |
| 31 | 48.53 | n.a. | 4.933 | 1.002 | 0.37 | n.a. |
| 32 | 49.42 | n.a. | 1.714 | 0.509 | 0.19 | n.a. |
| 33 | 51.73 | n.a. | 2.144 | 0.741 | 0.27 | n.a. |
| 34 | 52.83 | n.a. | 5.174 | 1.265 | 0.47 | n.a. |
| 35 | 53.96 | n.a. | 14.189 | 10.077 | 3.72 | n.a. |
| Total: | | | 997.492 | 270.753 | 100.00 | 7.141 |
