# Supplementary material for: An ethnopharmacological approach to evaluate antiparasitic and health-promoting abilities of Pueraria tuberosa (Willd.) DC. in livestock
Source: PLoS One. 2024 Jul 19;19(7):e0305667. doi: 10.1371/journal.pone.0305667 (PMC11259309; doi:10.1371/journal.pone.0305667)
Supplement: S3 Table — (PDF) [file pone.0305667.s003.pdf]

S3 Table: Quantification of selected phenolics and flavonoids from the methanolic extract of *P. tuberosa* tuber by HPLC analysis

Peak No.	Ret. Time	Peak Name	Height	Area	Ret. Area	Amount
	min		mAU	mAU*min	%	µg/ml
1	3.57	n.a.	12.518	2.496	0.28	n.a.
2	3.78	n.a.	19.075	2.949	0.33	n.a.
3	3.92	n.a.	206.823	12.667	1.41	n.a.
4	4.13	n.a.	67.804	15.860	1.77	n.a.
5	4.44	n.a.	5.799	0.606	0.07	n.a.
6	5.20	n.a.	19.939	2.378	0.27	n.a.
7	5.48	n.a.	12.965	2.526	0.28	n.a.
8	5.70	GALLIC ACID	15.081	1.711	0.19	1.506
9	5.95	n.a.	6.684	1.824	0.20	n.a.
10	7.13	n.a.	7.278	2.691	0.30	n.a.
11	8.90	n.a.	25.748	14.360	1.60	n.a.
12	11.34	CATECHIN	103.537	96.994	10.82	629.928
13	11.89	n.a.	1163.859	638.167	71.18	n.a.
14	12.99	n.a.	108.339	33.757	3.77	n.a.
15	13.91	n.a.	12.862	4.590	0.51	n.a.
16	14.70	n.a.	5.237	1.338	0.15	n.a.
17	15.32	n.a.	4.969	0.762	0.09	n.a.
18	15.69	n.a.	4.223	0.703	0.08	n.a.
19	16.53	n.a.	6.317	3.064	0.34	n.a.
20	17.46	n.a.	3.918	0.618	0.07	n.a.
21	18.02	n.a.	14.999	3.127	0.35	n.a.
22	18.90	n.a.	5.279	2.118	0.24	n.a.
23	20.56	NARINGIN	3.411	1.155	0.13	1.609
24	21.34	n.a.	9.524	4.128	0.46	n.a.
25	22.35	n.a.	9.611	2.017	0.22	n.a.
26	25.76	n.a.	10.401	3.925	0.44	n.a.
27	26.86	n.a.	30.350	10.778	1.20	n.a.
28	28.64	QUERCETIN				1.030
29	30.12	n.a.	3.358	1.544	0.17	n.a.
30	34.15	n.a.	2.897	0.668	0.07	n.a.
31	35.99	n.a.	3.150	0.854	0.10	n.a.
32	41.60	n.a.	10.394	3.244	0.36	n.a.
33	42.81	n.a.	4.675	1.422	0.16	n.a.
34	46.06	n.a.	2.695	0.633	0.07	n.a.
35	46.60	n.a.	11.243	6.201	0.69	n.a.
36	51.93	n.a.	3.264	0.935	0.10	n.a.
37	52.92	n.a.	4.013	1.103	0.12	n.a.
38	54.01	n.a.	16.924	12.661	1.41	n.a.
Total:			1959.163	896.572	100.00	634.073
