# Supplementary material for: An ethnopharmacological approach to evaluate antiparasitic and health-promoting abilities of Pueraria tuberosa (Willd.) DC. in livestock
Source: PLoS One. 2024 Jul 19;19(7):e0305667. doi: 10.1371/journal.pone.0305667 (PMC11259309; doi:10.1371/journal.pone.0305667)
Supplement: S4 Table — (PDF) [file pone.0305667.s004.pdf]

S4 Table: Quantification of selected phenolics and flavonoids from the ethanolic extract of *P. tuberosa* tuber by HPLC analysis

Peak No.	Ret. Time min	Peak Name	Height mAU	Area mAU*min	Ret. Area %	Amount µg/ml
1	3.58	n.a.	7.852	0.661	0.05	n.a.
2	3.80	n.a.	17.947	2.557	0.19	n.a.
3	4.05	n.a.	132.618	8.681	0.64	n.a.
4	4.25	n.a.	402.577	24.776	1.83	n.a.
5	4.60	n.a.	25.676	3.560	0.26	n.a.
6	6.09	GALLIC ACID	31.004	5.723	0.42	5.036
7	7.16	n.a.	7.120	2.888	0.21	n.a.
8	8.82	n.a.	31.451	28.304	2.10	n.a.
9	9.46	n.a.	36.320	14.728	1.09	n.a.
10	10.15	n.a.	76.436	18.892	1.40	n.a.
11	10.87	n.a.	144.618	23.854	1.77	n.a.
12	11.43	CATECHIN	121.250	22.428	1.66	145.662
13	11.85	n.a.	1501.117	1052.658	77.92	n.a.
14	12.76	n.a.	106.696	36.085	2.67	n.a.
15	13.88	n.a.	15.585	5.352	0.40	n.a.
16	14.67	n.a.	8.515	2.863	0.21	n.a.
17	15.29	n.a.	15.113	2.871	0.21	n.a.
18	15.65	n.a.	8.563	1.653	0.12	n.a.
19	16.01	n.a.	5.778	0.909	0.07	n.a.
20	16.48	n.a.	5.924	2.794	0.21	n.a.
21	17.44	n.a.	3.370	0.523	0.04	n.a.
22	17.99	n.a.	9.879	2.813	0.21	n.a.
23	18.86	n.a.	7.898	2.556	0.19	n.a.
24	20.48	NARINGIN	5.842	2.587	0.19	3.600
25	21.28	n.a.	8.550	3.637	0.27	n.a.
26	22.29	n.a.	11.734	4.705	0.35	n.a.
27	25.19	n.a.	8.855	3.720	0.28	n.a.
28	25.63	n.a.	14.020	6.363	0.47	n.a.
29	26.79	n.a.	50.763	20.537	1.52	n.a.
30	30.05	n.a.	5.852	2.533	0.19	n.a.
31	31.38	n.a.	1.382	0.932	0.07	n.a.
32	33.33	n.a.	3.038	1.331	0.10	n.a.
33	34.08	n.a.	5.128	1.091	0.08	n.a.
34	40.45	n.a.	3.908	1.342	0.10	n.a.
35	41.56	n.a.	17.488	5.950	0.44	n.a.
36	42.76	n.a.	6.076	2.400	0.18	n.a.
37	43.51	n.a.	2.830	1.303	0.10	n.a.
38	44.11	CURCUMIN	4.026	1.072	0.08	2.820
39	46.00	n.a.	5.616	1.192	0.09	n.a.
40	46.51	n.a.	11.383	4.847	0.36	n.a.
41	48.70	n.a.	5.751	1.619	0.12	n.a.
42	48.93	n.a.	5.544	1.538	0.11	n.a.
43	51.89	n.a.	6.198	1.512	0.11	n.a.
44	52.94	n.a.	6.083	1.929	0.14	n.a.
45	53.36	n.a.	5.253	1.194	0.09	n.a.
46	53.95	n.a.	14.082	9.512	0.70	n.a.
Total:			2932.708	1350.976	100.00	157.118
