# Supplementary material for: An ethnopharmacological approach to evaluate antiparasitic and health-promoting abilities of Pueraria tuberosa (Willd.) DC. in livestock
Source: PLoS One. 2024 Jul 19;19(7):e0305667. doi: 10.1371/journal.pone.0305667 (PMC11259309; doi:10.1371/journal.pone.0305667)
Supplement: S1 Fig — HPLC chromatogram of P. tuberosa tuber extracts: (A) Aqueous extract, (B) Methanolic extract, (C) Ethanolic extract. (PDF) [file pone.0305667.s005.pdf]

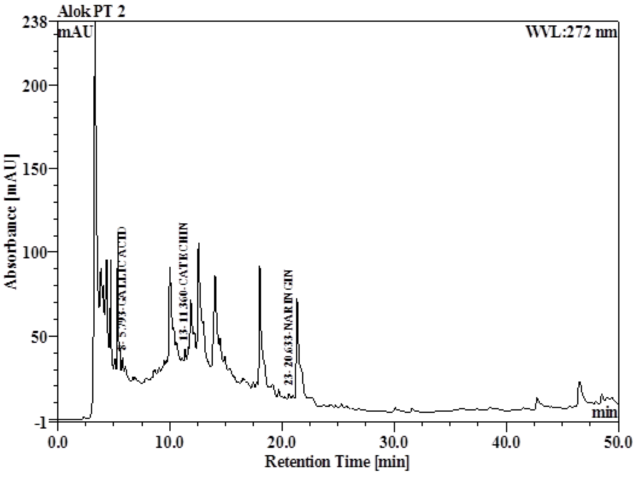

(A)

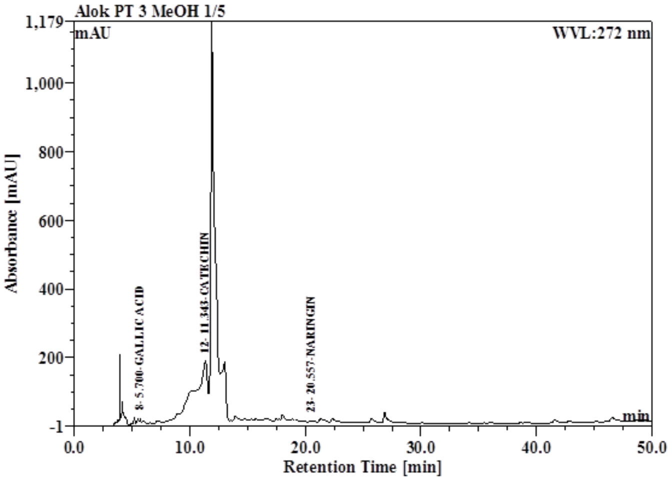

(B)

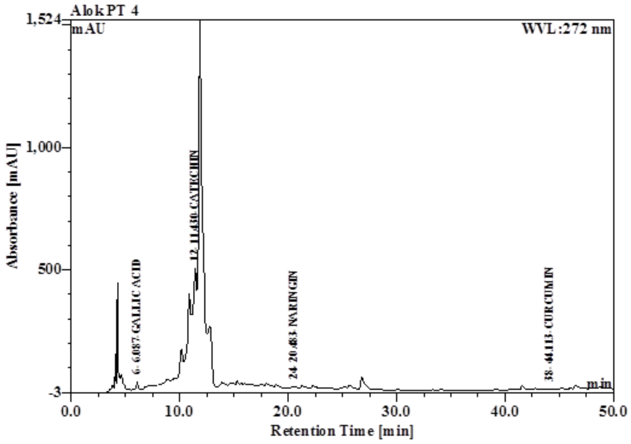

(C)

S1 Fig.

HPLC chromatogram of *P. tuberosa* tuber extracts: **(A)** Aqueous extract, **(B)** Methanolic extract, **(C)** Ethanolic extract.
