# Supplementary material for: An ethnopharmacological approach to evaluate antiparasitic and health-promoting abilities of Pueraria tuberosa (Willd.) DC. in livestock
Source: PLoS One. 2024 Jul 19;19(7):e0305667. doi: 10.1371/journal.pone.0305667 (PMC11259309; doi:10.1371/journal.pone.0305667)
Supplement: S2 Fig — A. GC-MS chromatogram obtained from methanolic extract of P. tuberosa; B. Chemical structures of the identified compounds. (PDF) [file pone.0305667.s006.pdf]

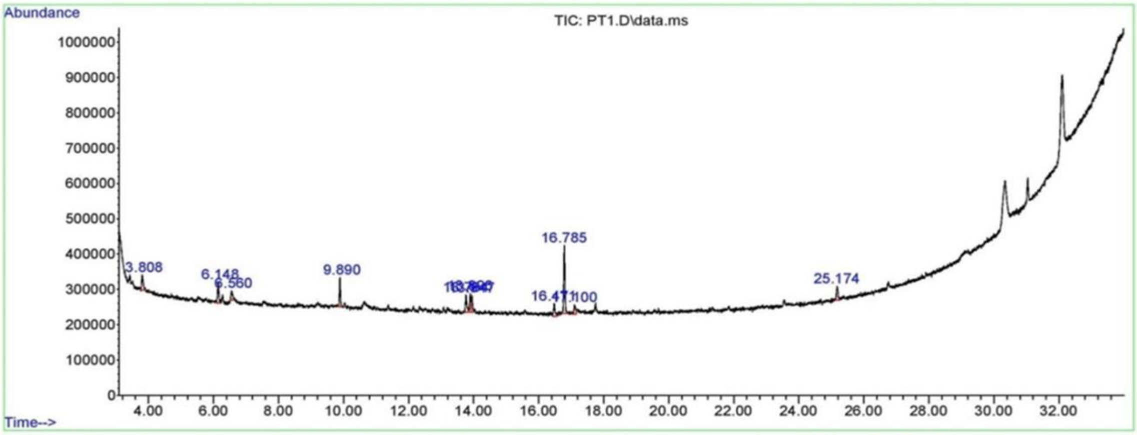

(A)

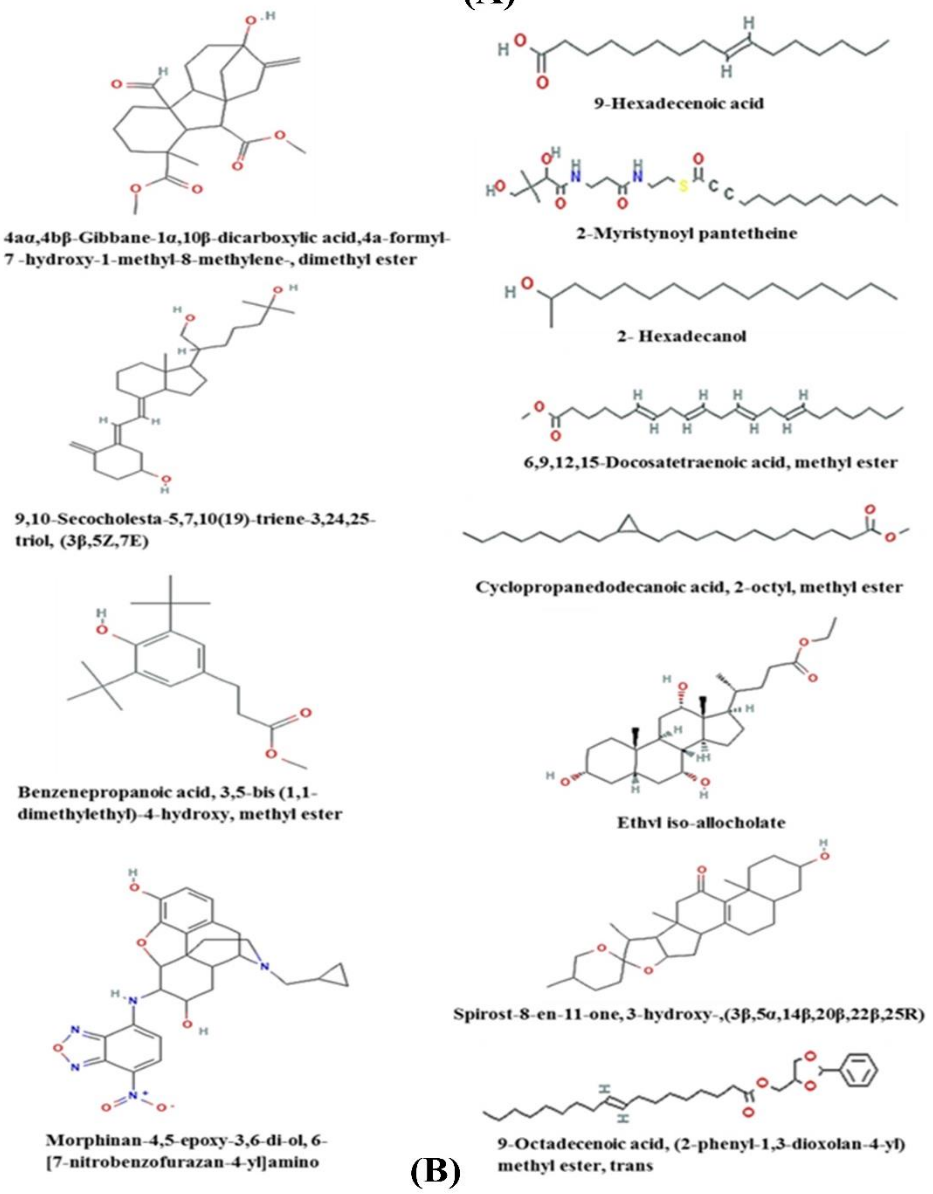

(B)

S2A Fig. GC-MS chromatogram obtained from methanolic extract of *P. tuberosa* tuber showing identified major peaks; **S2B Fig.** Chemical structures of the identified compounds (n=12) by GC-MS analysis of methanolic extract of *P. tuberosa* tuber.
